# Performance assessment of sample-specific network control methods for bulk and single-cell biological data analysis

**DOI:** 10.1371/journal.pcbi.1008962

**Published:** 2021-05-06

**Authors:** Wei-Feng Guo, Xiangtian Yu, Qian-Qian Shi, Jing Liang, Shao-Wu Zhang, Tao Zeng

**Affiliations:** 1 School of Electrical Engineering, Zhengzhou University, Zhengzhou, China; 2 Key Laboratory of Information Fusion Technology of Ministry of Education, School of Automation, Northwestern Polytechnical University, Xian, China; 3 Clinical Research Center, Shanghai Jiao Tong University Affiliated Sixth People’s Hospital, Shanghai, China; 4 Hubei Key Laboratory of Agricultural Bioinformatics, College of Informatics, Huazhong Agricultural University, Wuhan, China; 5 CAS Key Laboratory of Computational Biology, Bio-Med Big Data Center, Shanghai Institute of Nutrition and Health, University of Chinese Academy of Sciences, Chinese Academy of Sciences, Shanghai, China; Chinese Academy of Sciences, CHINA

## Abstract

In the past few years, a wealth of sample-specific network construction methods and structural network control methods has been proposed to identify sample-specific driver nodes for supporting the Sample-Specific network Control (SSC) analysis of biological networked systems. However, there is no comprehensive evaluation for these state-of-the-art methods. Here, we conducted a performance assessment for 16 SSC analysis workflows by using the combination of 4 sample-specific network reconstruction methods and 4 representative structural control methods. This study includes simulation evaluation of representative biological networks, personalized driver genes prioritization on multiple cancer bulk expression datasets with matched patient samples from TCGA, and cell marker genes and key time point identification related to cell differentiation on single-cell RNA-seq datasets. By widely comparing analysis of existing SSC analysis workflows, we provided the following recommendations and banchmarking workflows. (i) The performance of a network control method is strongly dependent on the up-stream sample-specific network method, and Cell-Specific Network construction (CSN) method and Single-Sample Network (SSN) method are the preferred sample-specific network construction methods. (ii) After constructing the sample-specific networks, the undirected network-based control methods are more effective than the directed network-based control methods. In addition, these data and evaluation pipeline are freely available on https://github.com/WilfongGuo/Benchmark_control.

## Author summary

Recently structural network control methods which exploit structural controllability of complex networks have become a powerful analysis tool for identifying driver nodes (e.g., driver genes or drug targets) in biological networked systems. In fact, the key point to apply structural network control on biological networked systems is how to characterize the state transition network (or interaction network) of these biological systems. The state transition network is a graph in which nodes denote the system variables and edges denote the significant interactions to trigger the state transition of system from one attractor (e.g., healthy state of individual patients) to another attractor (e.g., disease state of individual patients). The state transition network characterizes the state transition of networked system between any two attractors. Coincidentally, recently single sample network construction methods have been proposed to construct sample specific interaction network for characterizing the state transition of sample-specific biological systems. Therefore it is time to conduct a study to assess the performance of these state-of-the-art methods on the real-world biological systems, including sample-specific network construction methods and structural network control methods. Here we comprehensively provided a performance evaluation of 16 analysis workflows based on the combination of four sample-specific network reconstruction methods and four representative structural network control methods on a variety of real-world biological datasets. By this study, we have shed light on the relative behavior and performance of different analysis frameworks for evaluating and recommending these methods in particular biological application scenarios. In addition, we would like to point a few challenges for current methods and suggest several development directions of new SSC analysis workflows when more complex practice conditions of control principles are faced.

## Introduction

From dynamical system viewpoint, single samples can be modeled as sample-specific networked systems, of which the state transitions are determined by the sample-specific driver variables/nodes [1–6]. However, it is a big challenge for identifying sample-specific driver variables/nodes in dynamical biological processes called Sample-Specific network Control (SSC) problem, as the true functional form of the underlying dynamics for sample-specific biological systems are always unavailable. Recently structural network control approaches [7–11] which aim to find a minimum set of driver nodes for steering the states of large-scale networks to the desired states, may provide informative bioinformatics methods for solving the SSC problem [12–17]. Meanwhile, the analysis of SSC problem is dependent on the prior given network structure representing the topology of biological system. Indeed, how to obtain the network representation of one sample (e.g., one cell in single-cell studies or one tumor sample in precision medicine applications) is currently widely investigated [18–20]. Thus, lots of sample-specific network reconstruction methods and structural network control approaches have already been widely developed and are available for SSC analysis. Therefore, it is time to survey the SSC analysis workflows for the identification of sample-specific driver variables related to heterogeneous biological processes.

Obviously, SSC analysis workflow in single samples consists of two key steps: (i) constructing a sample-specific state transition network to characterize the state transition of each sample during dynamical biological processes; and (ii) designing network control methods based on the topological structure of the sample-specific state transition network. On one hand, several approaches have been proposed for constructing the sample-specific state transition network, including Single Pearson Correlation Coefficient (SPCC) [1,2], Linear Interpolation to Obtain Network Estimates for Single Samples (LIONESS) [3], Single-Sample Network (SSN) [4], and Cell-Specific Network construction (CSN) methods [5]. These sample-specific state transition networks represent gene pairs are involved in the biological process for each sample, and they are the key to applying follow-up structural network control methods. On the other hand, many structural network control approaches have also been developed, including two main categories. The first category as directed-network-based methods consists of Maximum Matching Sets based control methods (MMS) [7] and Directed Feedback Vertex based control method (DFVS) [21]. And the second category as undirected-network-based methods includes Minimum Dominating Sets based control method (MDS) [9] and Nonlinear Control of Undirected networks Algorithm (NCUA) [22].

Although these state-of-the-art methods are applicable in cutting-edge studies, they indeed have their pros and cons in solving detailed biological and biomedical problems due to the complicate application scenarios (e.g., incomplete available biological networks, unavailable reference samples, and non-determined association directions). However, no comprehensive evaluation of these algorithms has appeared. Apparently, guidelines or recommendations are required for biological and biomedical scientists to apply these workflows on SSC analysis efficiently; and the in-depth evaluation of these SSC analysis workflows is necessary to reveal their advantages and limitations on a variety of representative simulations and real-world datasets. Thus, in this work, we compared and evaluated multiple solutions/workflows for SSC applications in detail. First, we summarized four sample-specific network construction methods (i.e., SPCC, LIONESS, SSN, and CSN) and four structural control approaches (i.e., MMS, DFVS, MDS, and NCUA). Thus, we have included a total of 16 SSC analysis workflows based on the combination of any two methods corresponding to the two steps of SSC. Then we evaluated the usability of different analysis workflows on three kinds of datasets: (i) numerical simulation on two real biological networks; (ii) cancer driver gene and drug target identification on nine TCGA bulk gene expression datasets with matched normal and disease samples for individuals; and (iii) cell differentiation factor recognition on the temporal single-cell RNA-seq dataset. In these analysis and comparisons, the diverse evaluation measurements were carried out by using the F-measure for predicting cancer driver gene, the AUC for ranking drug combinations, the Jaccard score for method consensus and robustness, functional enrichment for biological significance, and so on.

Through this study, we found the SSC analysis workflows towards the final outcomes significantly depend on the characteristics of the network structure. Particularly, we summarized and provided several recommendations for these SSC analysis workflows. (i) The performance of a down-stream network control method is strongly dependent on the up-stream sample-specific network method, and CSN and SSN are two preferred sample-specific network construction methods according to a serious of evaluations. (ii) Overall, the undirected-network-based control methods (i.e., MDS and NCUA) are more effective than the directed-network-based control methods (i.e., MMS and DFVS) on most TCGA bulk cancer data and temporal single-cell RNA-seq data. These results demonstrate that the identified network driver nodes may be dominantly affected by the network characteristics (i.e., directed or undirected). In **Table 1**, we summarized all recommended methods in different evaluations. In addition, we provided freely available data and an evaluation pipeline, supporting biological context-specific recommendations for custom usage and benchmark study.

**Table 1:** The summary of recommended methods in different biological application scenarios. * denotes that the method is recommended in the SSC analysis.

Categories	Sample-specific network construction methods				Network control methods				Reference network		Recommendations from which Figure
ID	SPCC	LIONESS	SSN	CSN	MMS	MDS	DFVS	NCUA	Net-1	Net-2	
Simulated network								*	*		Fig 2
TCGA(driver genes)			*	*		*		*		*	Fig 3
TCGA(Drugs)			*	*		*		*		*	Fig 4
scRNA-seq				*				*		*	Figs 5–7

## Results

### Sample-specific network control problem and analysis workflows

**Fig 1:**
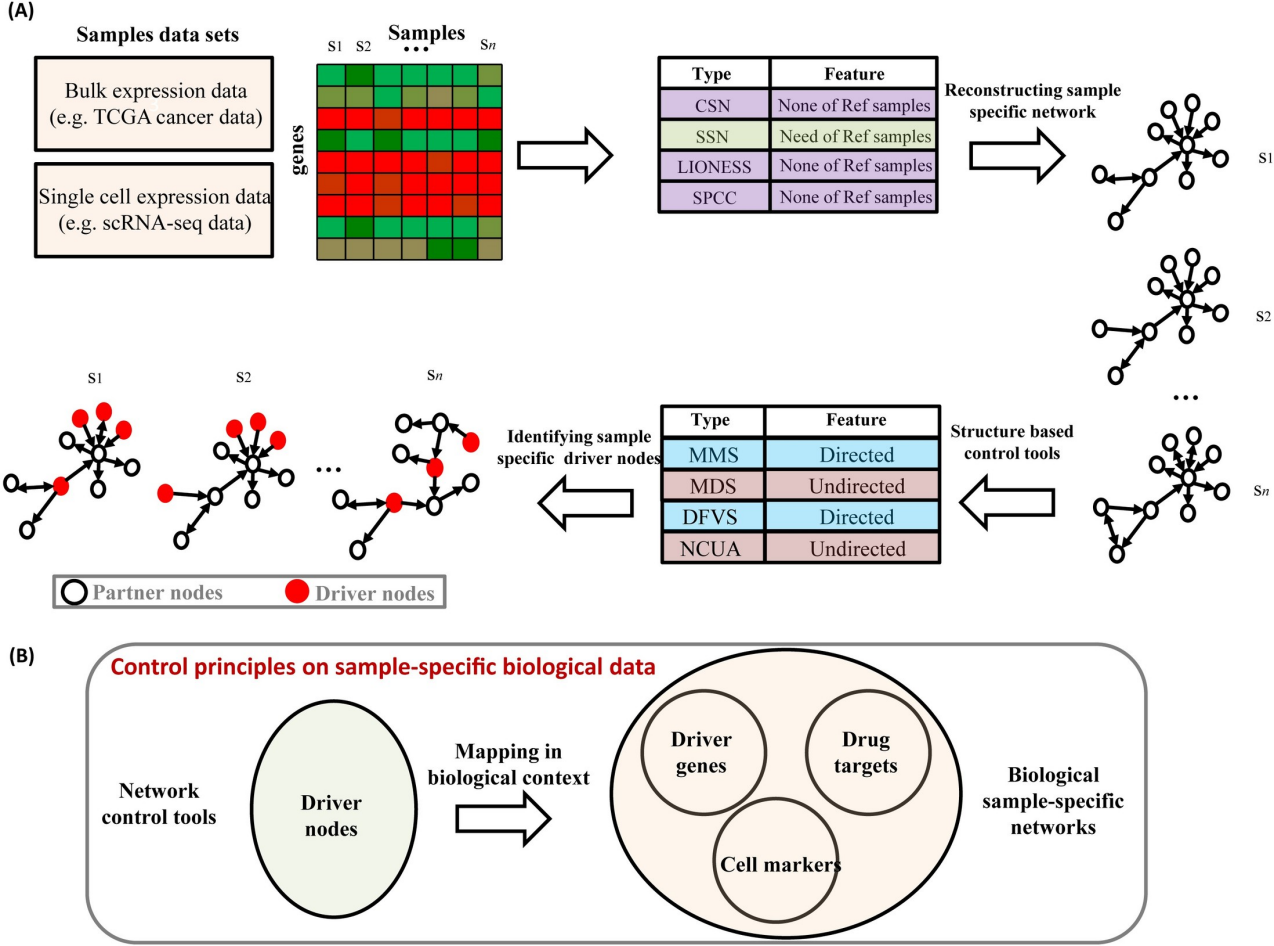
Overview of the Sample-Specific Control problem (SSC). (A) The flowchart of SSC analysis. The process of SCC analysis consists of two steps. The first step is to construct the sample-specific state transition network from the sample datasets. For constructing the sample-specific state transition network, several sample-specific network construction techniques have been proposed, including the Single Pearson Correlation Coefficient (SPCC), Linear Interpolation to Obtain Network Estimates for Single Samples (LIONESS), Single-Sample Network (SSN), and Cell-Specific Network construction (CSN) methods. Among them, the SSN method has the requirement for reference samples for constructing the single-sample differential co-expression network. Note that to filter the noise of sample-specific network reconstructions, the directed protein interaction information networks can be used for keeping the edge direction in the sample-specific state transition network. The second step is to design the network control principles; several structural network control methods have been proposed for finding a minimum set of driver nodes to control the whole network state dependent on adequate knowledge of the network structure, including the directed-network-based methods (MMS and DFVS) and the undirected-network-based methods (MDS and NCUA). (B) Representative biological meaning of “network driver nodes” in the structural network control principles. Assuming that biological samples can be represented as the sample-specific interaction network, the sample-specific network driver nodes can provide an efficient resource of personalized driver genes and cell-specific markers that can be useful for understanding the tumor or cell heterogeneity.

Given expression data of multiple samples, the goal of SSC analysis is to identify sample-specific driver variables for determining the sample state transition. The procedure of SSC analysis consists of two steps (**Fig 1A**). The first step is to construct the sample-specific state transition network, including several techniques such as SPCC [1,2], LIONESS [3], SSN [4], and CSN [5]. The state transition network is a graph in which nodes denote the system variables and edges denote the significant interactions to trigger the state transition of system from one attractor (e.g., healthy state of individual patients) to another attractor (e.g., disease state of individual patients). The state transition network characterizes the state transition potential of networked system between any two attractors. The second step is to identify network driver nodes (network drivers) in the network control principles, including directed-network-based network control methods (MMS [7] and DFVS [21]) and undirected-network-based network control methods (MDS [9] and NCUA [22]).

In this study, we constructed the sample-specific state transition networks by using sample-specific network construction methods on the sample gene expression data and prior known gene interaction data, thus they would also be called as sample-specific interaction networks in below. The edges of sample-specific interaction networks consist of the significant sample-specific differential co-expressions and prior known gene interactions. In this study, we used two prior-known biological directed gene/protein interaction networks. The first reference gene/protein interaction network was constructed by Hou et al. [23] (denoted as **Network-1**), which were integrated by a variety of recently curated database resources, such as MEMo [24,25], Reactome [26], NCI-Nature Curated PID [27], and KEGG [28]. Network-1 consists of 11,648 genes and 211,794 interactions. These interactions consist of gene-interaction information from multiple high-quality sources such as protein interactions, TF-target interactions, protein domain interactions, text-mined interactions, etc. The second reference gene/protein interaction network consists of 6339 genes/proteins and 34,813 directed edges (denoted as **Network-2**), constructed by Vinayagam et al. [29]. Of note, we considered the gene co-expression network as a bidirected network, where the directionality of one network edge denotes the connection for which two genes in this edge are both highly or both lowly expressed. Thus, the biological interpretation of sample-specific interaction networks indicates the significant change of sample-specific gene interactions on co-expression level.

Actually, the sample-specific interaction network can reliably characterize the biological system or sample state. In a biological context, the network drivers in such sample-specific network (i.e., SSC solution) can be, for example, the driver genes underlying the cancer development or cell marker genes underlying single cell identification (**Fig 1B**). Therefore, the sample-specific network driver nodes can serve as an efficient resource of personalized driver genes or cell type/state specific markers, which can deepen our understanding on tumor or cell heterogeneity. Here we focused on the task of applying SSC workflow suitably which combined sample-specific network construction and structural network control methods for the identifications of sample-specific driver variables related to particular biological processes, in the method comparison and evaluation manner. In this study, we defined the minimum driver nodes to satisfy the controllable of networks as driver nodes in a network by using network control methods. Indeed, driver node sets are usually not unique for network control methods such as MMS, MDS, DFVS and NCUA. Generally, MDS and DFVS does not consider multiple driver-node sets to control the network. In order to fairly compared these control methods, we choose one minimum driver nodes set as the driver node set in a given sample specific network for the following analysis.

### Evaluation of network control methods by pinning control simulation on biological networks

To evaluate the physical significance of structural network control methods on biological data ahead of SSC workflow evaluation, we focused on the **control efficiency,** which measures the performance of structure network control methods on networked system. To avoid the influence of network completeness and confidence, we firstly synthetically applied a three-dimensional stable nonlinear Lorentz oscillator system [30,31] with topological structures from two biological directed protein interaction networks (i.e., Network-1 and Network-2). That is, all nodes are considered as the nonlinear Lorentz oscillator systems and all nodes are connected as those in the directed protein interaction networks. The Lorentz oscillator networked system can be modeled mathematically as,

$$\dot{x_{i}(t)}=\left[\begin{array}{l} \dot{x_{i1}(t)}\\ \dot{x_{i2}(t)}\\ \dot{x_{i3}(t)} \end{array}\right]=\left[\begin{array}{l} a(x_{i2}(t)-x_{i1}(t))+c\sum_{j=1}^{N}a_{ij}x_{j}(t)\\ qx_{i1}(t)-x_{i1}x_{i3}(t)-x_{i2}(t)+c\sum_{j=1}^{N}a_{ij}x_{j}(t)\\ x_{i1}x_{i2}(t)-bx_{i3}(t)+c\sum_{j=1}^{N}a_{ij}x_{j}(t) \end{array}\right],$$ (1)

where *a* = 10, *b* = 8/3, *q* = 28, *A* = (*a*_*ij*_)_*n*×*n*_ denotes the adjacency matrix of the network (i.e. simulated from Network-1 and Network-2) and such system has three stable attractors, i.e., (0,0,0), (8.484,8.484.27) and (-8.484,-8.484.27).

Then we identified minimum driver node sets by using network control methods (i.e., MMS, MDS, DFVS and NCUA), and we applied feedback controllers on the driver nodes to control the state of all nodes in the gene interaction networks from any initial state towards the desired attractor. The feedback controllers are defined as follows,

$$u_{i}=-k(x_{i}(t)-\bar{x}),\;i\in D\subseteq G$$ (2)

where *k* is the control strength (here we set *k* = 1000); *x*_*i*_(*t*) is the sate of node *i* at time *t*; $\bar{x}$ is the desired attractor; *D* denotes the set of driver nodes, *G* represents the gene interaction network. If the error between the final state and the desired state for given nodes is less than a tolerance error *ε*_0_, i.e., $\varepsilon =\left\|x_{i}(T)-\bar{x}\right\|\leq \varepsilon_{0},i\in G$ (*T* is the final time), we considered these nodes as efficiently controlled nodes. The control efficiency on whole network can be defined as *Efficiency* = ‖*E*‖/‖*D*‖, where *E* is a set of efficiently controlled nodes and *D* is a set of network driver nodes. The bigger the efficiency score is, the better the network control method performs.

**Fig 2:**
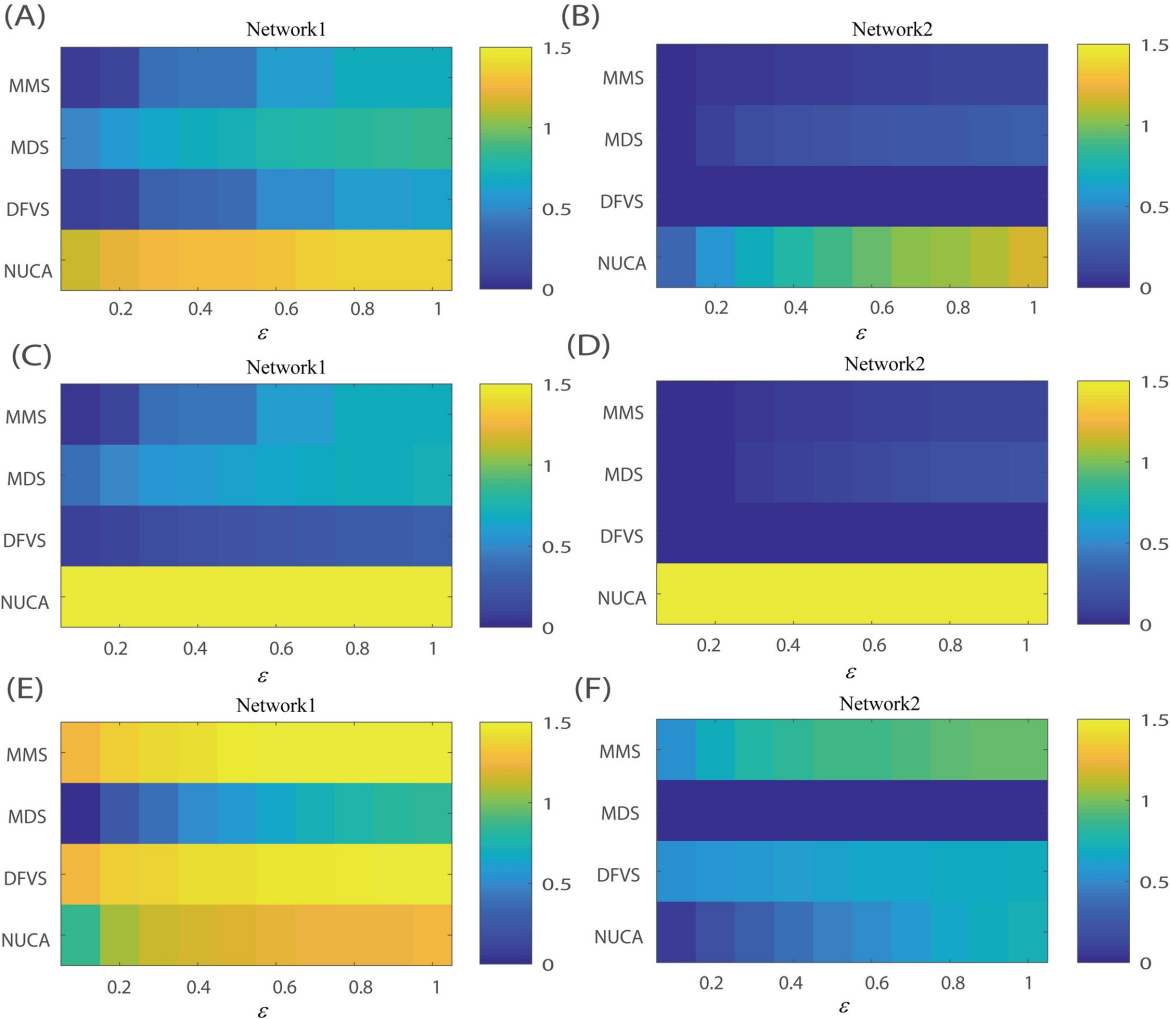
The evaluation of structural network control methods by simulation of biological networks. The goal is to evaluate the control efficiency by synthetically achieving control of the networked nonlinear Lorentz oscillator system towards desired attractors on two biological directed protein interaction networks from different resources (i.e. Network-1 and Network-2). (A-B) Desired attractor (8,484, 8.484, 27); (C-D) Desired attractor (-8,484, -8.484, 27); (E-F) Desired attractor (0, 0, 0). The efficiency of a given network is measured by the average value for different tolerance errors from 0.1 to 1.

Finally, we chose three attractors as the desired attractor respectively and conducted our network control methods as above procession to evaluate the effect of different desired attractors on the control efficiency of network control methods. The control efficiency results of different network control methods on Network-1 and Network-2 respectively were shown in **Fig 2,** where the results are obtained under different error tolerances being 0.1:0.1:1. It was found that NCUA performs better than other methods on control efficiency for attractor (8,484, 8.484, 27) and attractor (-8,484, -8.484, 27). For attractor (0, 0, 0), NCUA, MMS and DFVS perform better than MDS. Overall, NCUA has stable performance for different desired attractors no regardless of sample specific network construction methods and prior know interaction networks were used. Furthermore when Network-1 were used, the performance of NCUA is robust for different desired attractors no regardless of sample specific network construction methods networks were used. Therefore, NCUA would be recommended for pinning control analysis on these two biological networks. And the total recommendations were summarized as shown in **Table 1**.

### Evaluation of SSC by cancer driver gene and drug target identification on TCGA bulk gene expression data

To demonstrate the usefulness of SSC analysis, we applied 16 SSC analysis workflows on 9 TCGA cancer datasets to discover the personalized driver genes and personalized drug targets. They are the datasets for breast invasive carcinoma (BRCA), colon adenocarcinoma (COAD), kidney chromophobe (KICH) and kidney renal clear cell carcinoma (KIRC), kidney renal papillary cell carcinoma (KIRP), liver hepatocellular carcinoma (LIHC), lung adenocarcinoma (LUAD), lung squamous cell carcinoma (LUSC), and uterine corpus endometrial carcinoma (UCEC). The paired or matched samples for each individual patient (i.e., a normal sample and a tumor sample from the same patient) were obtained from the TCGA data portal. The detailed information of cancer samples used in this study is summarized in **Table A in S1 File**.

For each individual patient, we applied the CSN, SSN, SPCC, and LIONESS models to obtain the personalized gene state transition network (i.e. sample-specific gene interaction network). Of note, SSN uses a paired version by using both tumor sample and normal sample information, but CSN, SPCC, and LIONESS only use tumor sample information for constructing the personalized state transition network (**Materials and Methods**). To filter the noise of sample-specific co-expression networks, we used two directed gene/protein interaction information networks (i.e., the references from **Network-1** and **Network-2**) to keep the edge direction, namely, CSN_Net1, CSN_Net2, SSN_Net1, SSN_Net2, SPCC_Net1, SPCC_Net2, LIONESS_Net1, and LIONESS_Net1. Then, using these eight personalized state transition networks constructed in the first step of SSC, the structural network control methods, i.e., the MMS-based control method (full control), MDS-based control method, and FVS-based control methods (DFVS and NCUA), further identified sets of key genes as the potential personalized driver genes or drug targets.

**Fig 3:**
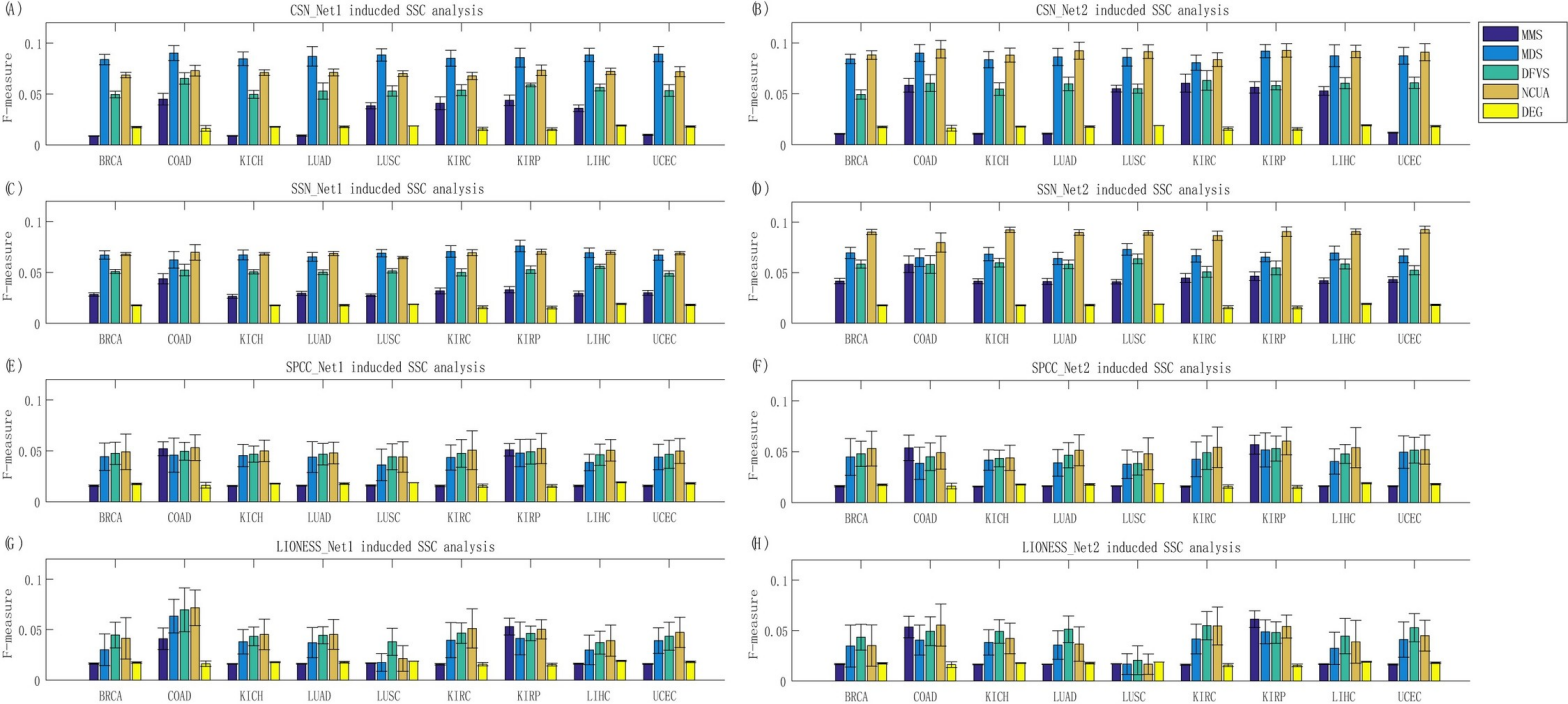
Evaluation of SSC workflows for driver gene identification on nine TCGA bulk cancer gene expression datasets. By using dissimilar combinations of sample-specific network and reference network, different sample-specific state transition networks can be obtained, e.g., (A) CSN_Net1, (B) CSN_Net2, (C) SSN_Net1, (D) SSN_Net2, (E) SPCC_Net1, (F) SPCC_Net2, (G) LIONESS_Net1, and (H) LIONESS_Net2. Then the performance of four network structural control methods based on these sample-specific state transition networks were evaluated, representing the performances of different SSC analysis workflows.

**Fig 4:**
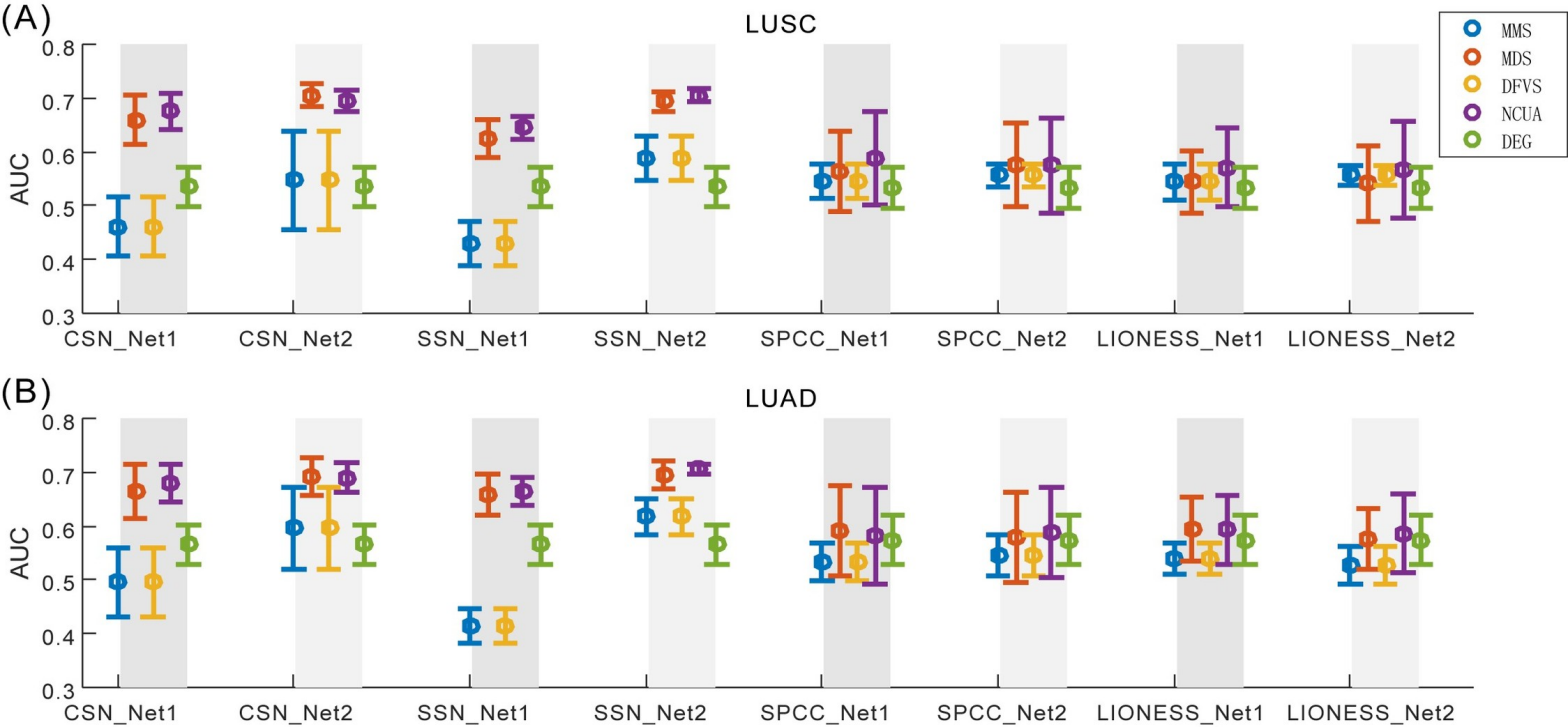
Evaluation of SSC workflows for drug target identification on LUSC and LUAD cancer datasets from TCGA. To evaluate the usage efficiency of sample-specific network drivers for personalized drug target discovery, the number of targeted sample-specific network drivers matching with drug combinations are calculated and anti-cancer drug target combinations are ranked for each patient. The drug combinations annotated in the Clinical Anti-cancer Combinational drugs (CAC) were applied to assess the AUC of the top-ranked/predicted anti-cancer drug combinations from different SSC workflows using (A) LUSC and (B) LUAD cancer datasets.

On one hand, to verify the effectiveness of these SSC solutions based on different structural network control methods, the prior-known cancer genes annotated in the list of Cancer Census Genes (CCGs) were applied to assess the F-measure of the predicted personalized driver genes (see **Materials and Methods**) considering both the precision and the recall). The results in terms of F-measure are listed in **Fig 3,** while the results in terms of precision and recall were listed in **Figs A and B** of **S1 File**, respectively. On the other hand, to evaluate the efficiency of sample-specific network drivers recognition for personalized drug target discovery, the number of sample-specific network drivers/genes targeted by drug combinations were calculated, and anti-cancer drug combinations were ranked for each patient based on the combinational drug target network **(S2 File)**. The drug combinations annotated in the Clinical Anti-cancer Combinational (CAC) drugs (**S3 File**) were applied to assess the AUC of the top-ranked anti-cancer drug combinations recommended by different SSC workflows (see **Materials and Methods**), and the representative results of LUSC and LUAD are shown in **Fig 4.**

From these results shown in **Figs 3** and **4**, we have several observations and conclusions. (i) Compared to the traditional Differential Expressed Gene (DEG) method, CSN and SSN would be more effective to support follow-up network structure control methods for discovering personalized driver genes (**Fig 3**) and predicting personalized combinational drug targets (**Fig 4**). And the undirected-network-based control methods (MDS and NCUA) are more effective than the directed-network-based control methods (MMS and DFVS) regardless of the sample-specific network construction method used. These results matched well with the experimental results in our above synthetic pinning control evaluations (**Fig 2**). (ii) Actually, the choice of proper prior-given network structure is also an important factor for SSC analysis. For example, in **Fig 3**, the F-measure of NCUA for SSN_Net2 is larger than that for SSN_Net1; and in **Fig 4**, the AUC of four network control methods based on CSN and SSN indeed exhibit better performance for the prediction of personalized combinational drug targets when Network-2 was used than those when Network-1 was used. These results matched well again with our synthetic pinning control evaluations (**Fig 2**), demonstrating that the choice of a proper prior-given interaction network structure is a key for applying SSC analysis in research and applications. By summarizing these results, CSN and SSN and reference Network-2 are recommended for supplying a sample-specific network; and MDS and NCUA are recommended as network control methods for discovering (targeted) network drivers, which were also listed in **Table 1**.

To evaluate the effect of number of reference samples on the performance of these sample-specific network construction methods on TCGA cancer data, we have randomly chosen 10%, 30% and 50% patients with paired normal and disease samples, and re-conducted the CSN, SSN, LIONESS and SPCC on these selected patients for each disease with similar procedure as above analysis and discussion, where this procedure was repeated 100 times. Comparing these average rates of F-scores from new SSC analysis on selected samples and those from original SSC analysis on all samples, it was found that the size of reference samples has smaller effect on the performance of CSN, compared with other network construction methods (**Figs C and D in S1 File**).

To evaluate whether network deconvolution [32] benefits for the performance improvement on SSC analysis, we firstly chose BRCA cancer data with relatively large number of patients (>100 paired samples of patients) among above nine TCGA cancer datasets for the further SSC analysis. Then we compared the results of SSC analysis with network deconvolution and without network deconvolution on BRCA cancer dataset, where network deconvolution can help identify and remove spurious transitive edges from co-expression network due to indirect effects. Comparing the average rates of F-scores of new SSC analysis with network deconvolution and those of original SSC analysis without network deconvolution on Network-1 and Network-2, CSN is better than others (**Fig E** in **S1 File**), which suggest that network deconvolution can more effectively improve the performance of SSC analysis when CSN was used.

### Evaluation of SSC by key factor recognition from temporal single-cell gene expression data

**Fig 5:**
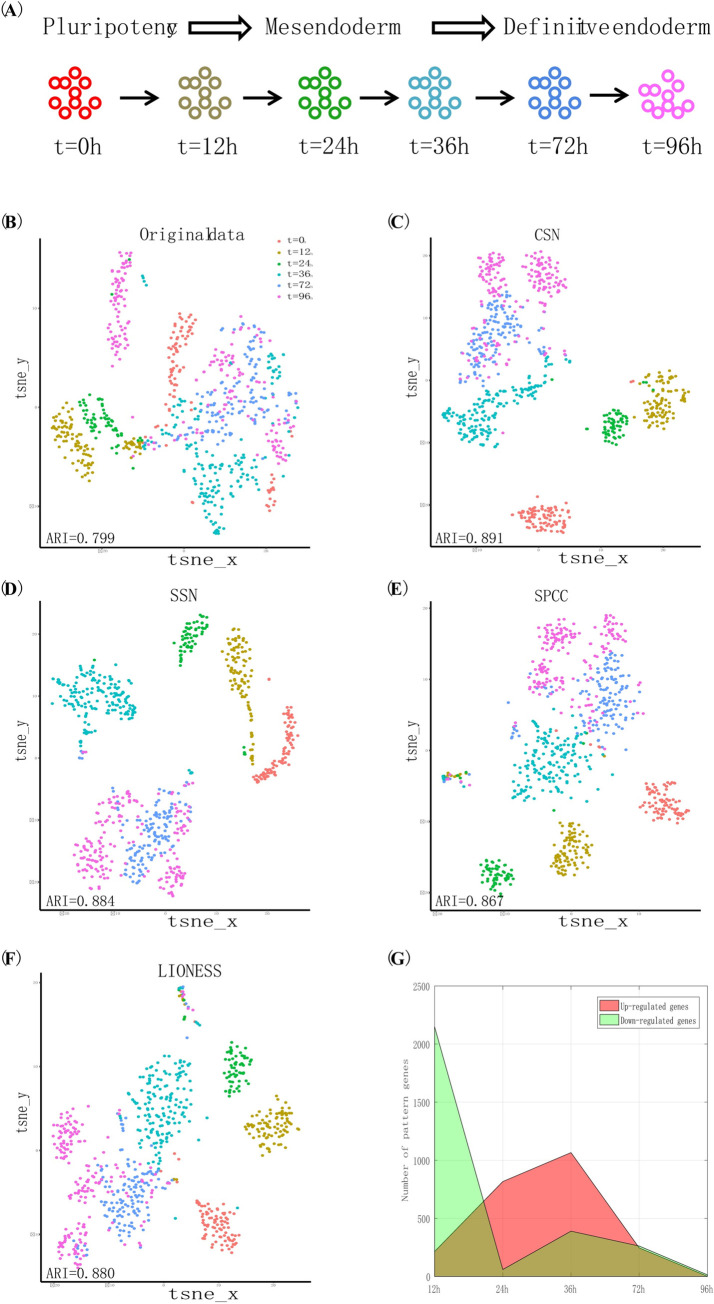
Evaluation of SSC workflows by key genes identification on Chu-time single-cell gene expression dataset. (A) Introduction of the Chu-time dataset. This dataset comes from a study of developmental biology and contains 758 cells within six time points (0 h, 12 h, 24 h, 36 h, 72 h, and 96 h) along the differentiation process from human embryonic stem cells to definitive endoderm cells. (B)–(F) The usage of four sample-specific network construction methods in identifying the cell clusters, i.e. cell types/states. (G) Detection of a group of genes with significant expression changes along the multiple time points as determined by SCPattern method.

As another kind of performance evaluation of different SSC workflows, 16 SSC analysis workflows were also carried on the Chu-time dataset [33]. This dataset comes from a study of developmental biology, containing 758 cells within six time points (0 h, 12 h, 24 h, 36 h, 72 h, and 96 h) along the differentiation process to produce definitive endoderm cells in human embryonic stem cells, as illustrated in **Fig 5A**. We first evaluated four network control methods in identifying the cell type appearance during the cell differentiation process from the stemness state to the differentiated state. Notably, a gene network-degree matrix rather than original gene expression matrix was applied for cell type identification (e.g., cell clustering). This new data matrix, in which each element is not the gene expression level, but the number of edges connected to each gene in the corresponding sample-specific interaction network, was constructed for CSN, SSN, SPCC, and LIONESS respectively. As the classification label (i.e., cell type) of each cell was known from the previous study [33], the adjusted random index (ARI) [34] was used as the performance measurement in method comparisons. Seeing **Fig 5B**, four sample-specific network construction methods are able to reconstruct the ordered clusters of single cells corresponding to the differentiation stages, and they may produce better results than those on the original data profiles (i.e., higher ARI values were obtained from gene network-degree matrix than those from gene expression data).

**Fig 6:**
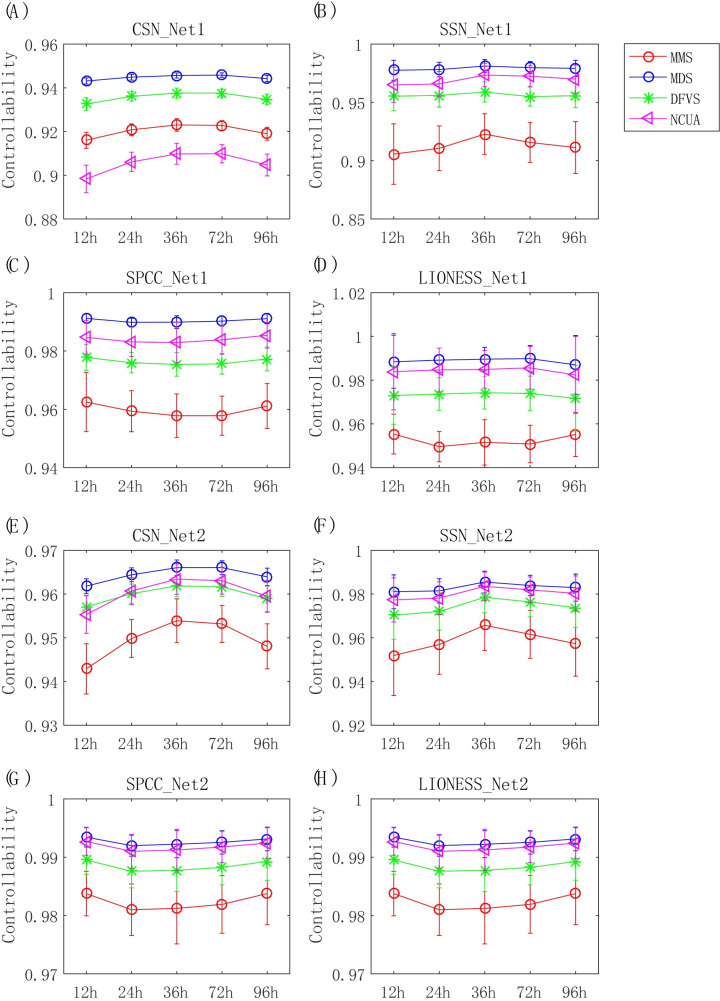
Evaluation of SSC workflows by the identification of important genes involved in cell fate decisions. The corresponding SSC analysis dependent on different sample-specific state transition networks, including (A) CSN_Net1, (B) SSN_Net1, (C) SPCC_Net1, (D) LIONESS_Net1, (E) CSN_Net2, (F) SSN_Net2, (G) SPCC_net2, and (H) LIONESS_Net2.

Next, from the SCPattern analysis [35] of the Chu-time dataset, as shown in **Fig 5C**, we detected a group of genes with expression changes along the different consecutive conditions (Up-regulated genes and Down-regulated genes); we found that the number of Up-regulated genes peaks at 36 h, implying that the key time point during embryonic differentiation may be around 36 h. To further validate this conclusion, we applied network structural control methods of SSC for evaluating the *controllability* of each cell (see **Materials and Methods**) at each time, which indicates the ability of each cell to change its current state; the bigger the *controllability* of one cell is, the greater the probability this cell’s state will change. As seen from **Fig 6**, the controllability remains unchanged or changes non-monotonically under different methods with SPCC and LIONESS and cannot find any significant key time point, regardless of the reference network applied. Meanwhile, all of the network structural control methods using CSN_Net2 detected the key time point at 36 h during cell differentiation according to the change of cell controllability, which would be consistent with the change of up-regulated genes detected by SCPattern. These results demonstrated that CSN and reference Network-2 are recommended for supplying sample-specific interaction networks for follow-up network control methods on such single-cell data (**Table 1**).

Moreover, the important genes for each cell can be identified by SSC workflows. As known, the rapid advancement of single-cell technologies has shed new light on the complex mechanisms of cellular heterogeneity, however, single-cell RNA-seq (scRNA-seq) suffers from higher noise and lower coverage compared to bulk RNA sequencing (RNA-seq). Based on statistical independence, cell-specific network would be able to quantify the overall reliable interactions between genes for each cell [5]. Thus, on the basis of cell-specific or sample-spcific networks, network control methods were applied to identify important functional genes of a single cell during human embryonic differentiation. We collected 20 functional genes that are involved in human embryonic differentiation (**S4 File**) from literature reports, and we applied the hypergeometric test [36] (see **Materials and Methods**) for computing the *P*-value of enrichment of identified important genes in those prior-known functional genes. The cells with a *P*-value less than 0.05 are considered as control-efficient cells, whose biological meaning points these cells are involved in human embryonic differentiation. **Fig F** in **S1 File** illustrated the number of control-efficient cells at different time points during embryonic differentiation.

When holding an assumption that the number of control-efficient cells should have much higher value during the differentiation process, the network control methods based on SPCC and LIONESS identified much smaller fraction of control-efficient cells at different time points compared with CSN and SSN. These results demonstrated that CSN and SSN would be the better options for sample-specific network construction in SSC analysis on this single-cell data. Furthermore on the CSN and SSN provided sample-specific interaction networks, MDS and NCUA exhibited better performance and robustness than other network control methods, regardless of the reference network applied. Therefore, CSN and reference Network-2 are recommended for supplying a sample-specific network construction, and MDS and NCUA are recommended as network control methods on single-cell data analysis. These consistent recommendations are supported by the results in **Figs 5** and **6,** and **Fig F in S1 File**, which are also summarized in **Table 1**.

### Evaluation of SSC by identification of “dark genes”

**Fig 7:**
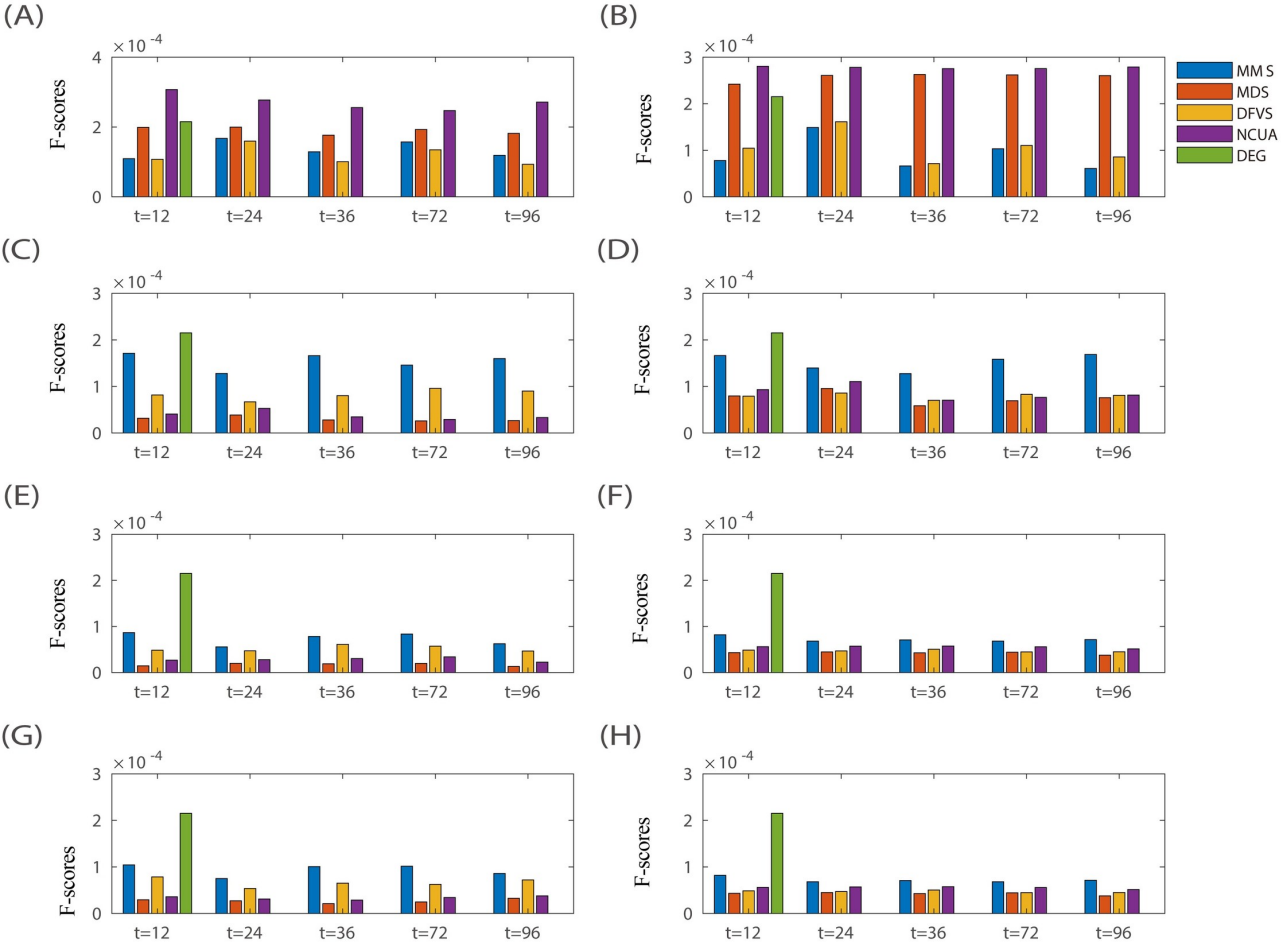
Identification of “dark genes”by different SSC workflows on Chu-time single cell dataset. The different state transition networks include (A) CSN_Net1, (B) CSN_Net2, (C) SSN_Net1, (D) SSN_Net2, (E) LIONESS_Net1, (F) LIONESS_Net2, (G) SPCC-Net1, and (H) SPCC_Net2.

To demonstrate whether SSC is able to detect important genes within dark gene regions, we have collected the genes within dark gene regions from a canonical dataset [37], which were regarded as dark genes for method evaluation in this study (**S5 File**). The results in terms of the ability to find “dark genes” in nine TCGA cancer data sets were shown in **Fig G** in **S1 File**, and the results of “dark genes” detected in Chu-time data set is shown in **Fig 7**. In brief, the ability of SSC analysis to identify “dark genes” is greatly dependent on datasets and sample-specific network construction methods.

(i) On multiple TCGA cancer datasets (**Fig G** in **S1 File**), MMS has a similar ability to identify “dark genes” as Differential Expression Genes (DEG) selection method for a given network construction method and a given dataset. However, MDS, DFVS and NCUA have less ability to identify “dark genes” than Differential Expression Genes (DEG) selection method.

(ii) Meanwhile, for the Chu-time single cell dataset, the final performance of SSC analysis is dependent on the choice of sample-specific network construction methods and the reference network (**Fig 7)**. In particular, NCUA exhibited better performance when CSN_Net1 was used. Thus, CSN and reference Network-1 and NCUA are recommended to combine as a SSC workflow to identify “dark genes” on Chu-time dataset.

Besides, the above discussions demonstrate that SSC is able to detect potential driver genes with important roles in the biological network control, meanwhile, the importance of driver genes without differential expression would be underestimated by conventional analysis on the expression level, so that they could also be thought of as “dark-differential expression genes”, e.g. on transcriptome level [2,5]. It is necessary to further evaluate whether different SSC analysis workflows have particular abilities or preferences to detect such “dark-differential expression genes”. For nine TCGA cancer datasets, we obtained the personalized “dark-differential expression genes” by calculating the fold change of driver genes’ expressions between normal and tumor samples (|log_2_(fold change)|<1). For the Chu-time dataset [33], we applied the SCPattern method [35] for detecting a group of “dark-differential expression genes” along differentiation time points.

**Fig 8:**
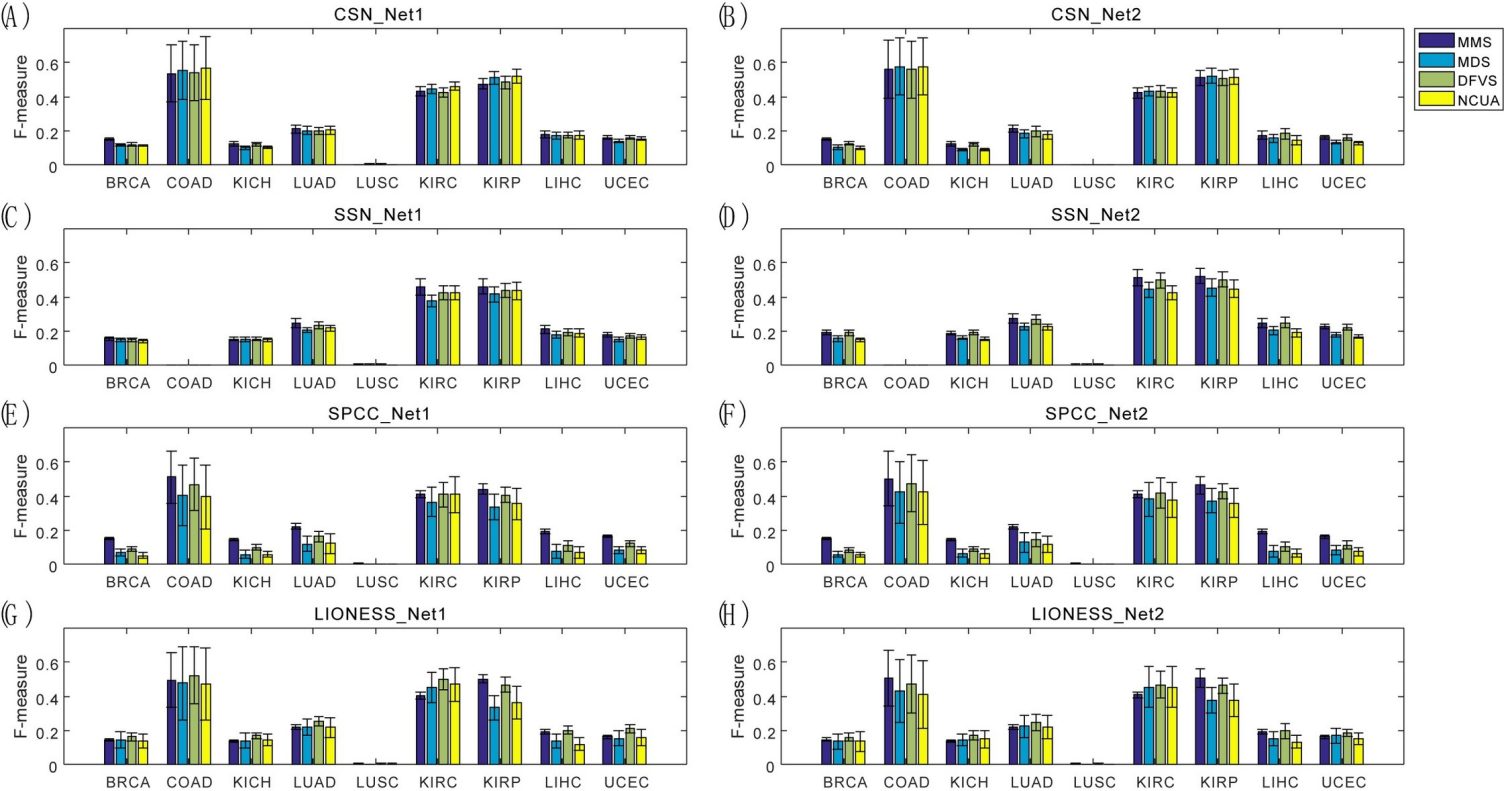
Identification of “dark-differential expression genes” by different SSC workflows on multiple TCGA cancer expression datasets. The different state transition networks includes (A) CSN_Net1, (B) CSN_Net2, (C) SSN_Net1, (D) SSN_Net2, (E) SPCC_Net1, (F) SPCC_Net2, (G) LIONESS_Net1, and (H) LIONESS_Net2.

The results in terms of the ability to find “dark-differential expression genes” (e.g. F-measure) on nine TCGA cancer data sets were shown in **Fig 8** while the results of Chu-time data set is shown in **Fig H in S1 File**. Generally, the ability of SSC analysis to identify “dark-differential expression genes” is greatly dependent on datasets and sample-specific network construction methods. (i) In multiple TCGA cancer datasets, for a given network construction method and a given dataset, all of the network control methods have a similar ability to identify “dark-differential expression genes” (**Fig 8**). (ii) Meanwhile, for the Chu-time dataset, as shown in **Fig H in S1 File**, the final performance of SSC analysis is dependent on the choice of sample-specific network construction methods and the reference network. In brief, NCUA exhibited better performance when CSN_Net1 was used and MMS performs better when CSN_Net2 and SSN (SSN_Net1 and SSN_Net2) and LIONESS (LIONESS_Net1 and LIONESS_Net2) and SPCC (SPCC_Net1 and SPCC_Net2) were used. By summarizing the above results, CSN and reference Network-1 and NCUA would be recommended as combined SSC workflow to identify “dark-differential expression genes”. Thus, when using SSC analysis on a particular dataset in practice, the users should evaluate the sample-specific network control methods and network structural control algorithms to balance the identification of potential “dark-differential expression genes” and conventional DEGs.

## Note 1: Significant SSC analysis rather than random selection

i) To demonstrate the performance of SSC, we used z-score to evaluate whether the performance of SSC analysis performs better than that of the random selection method and degree-preserved random selection method on TCGA cancer datasets. Without loss of generality, we choose BRCA cancer data with relatively large number of patients among multiple TCGA cancer datasets for the identification and discussion of driver genes and dark genes. Of note, the dark genes were regarded as the genes within dark gene regions from a canonical dataset [37]. Similarly, we chose LUAD cancer data with relatively large number of patients among LUNG (i.e., LUAD and LUSC) cancer datasets for the identification of drug combinations. The random selection method randomly selected the genes with the same number of SSC selected and we repeated this procedure 100 times. The degree-preserved random selection method generated 100 topologically matched random networks, each of which maintains the degree distribution of the original sample-specific interaction network used in SSC analysis. The z-score is defined as follows.

$$z_{i}=(n_{i}-mean(SD_{i}))/(std(SD_{i}))$$ (3)

where *n*_*i*_ is the result of SSC analysis for sample *i*, *SD*_*i*_ is the distribution of the distribution of the result of random selection method or degree-preserved random selection method. For this one-side statistic test, the null hypothesis (*H*_0_) is *n*_*i*_ = *n*_*0*_ and the alternative hypothesis (*H*_1_) is *n*_*i*_ > *n*_*0*_, where *n*_*0*_ is mean value of *SD*_*i*_. If z-score is greater than 1.645 (i.e., P-value <0.05), we can regard that the SSC analysis performs better than the random selection method or the degree-preserved random selection method.

We compared two random selection methods with SSC analysis by above reported results, including: (1) the z-score results of SSC analysis compared to random selections in terms of the F-score for CGC gene enrichment on BRCA cancer data, shown in **Fig I** in **S1 File;** (2) the z-score results of SSC analysis compared to random selections in terms of the F-score for clinical efficient combinational drug target enrichment on LUAD cancer data, shown in **Fig J in S1 File;** (3) the z-score results of SSC analysis compared to random selection in terms of the F-score for enriching in dark genes, shown in **Fig K in S1 File**.

By summarizing the results in **Figs I-K in S1 File**, the performance of MDS and NCUA on the sample-specific network of CSN and SSN performs better than those of random selection methods. And the results of MDS method on the sample-specific network of CSN perform better than that of degree-preserved random selection method. Therefore, SSC workflows actually can have significant findings rather than random selections.

ii) To evaluate whether the performance of SSC analysis performs better than that of two random selection methods on Chu-time single cell data, we defined the *significance score* of SSC as the average ratio between number of efficient cells of SSC analysis and those of the random selections. Obviously, in such a fold-change evaluation manner, we can regard that the SSC analysis performs better than the random selections when the *significance score* is greater than 1. As shown in **Figs L and M in S1 File,** most SSC analysis has significant discoveries rather than random selections. Meanwhile, the results of network control methods on the sample-specific network of SSN, LIONESS and SPCC performs better than that of degree-preserved random selection method. These results further enhance the conclusion that SSC workflows actually can have significant findings rather than random selections.

## Note 2: Consensus on identified driver genes between different network structural control methods

**Fig 9:**
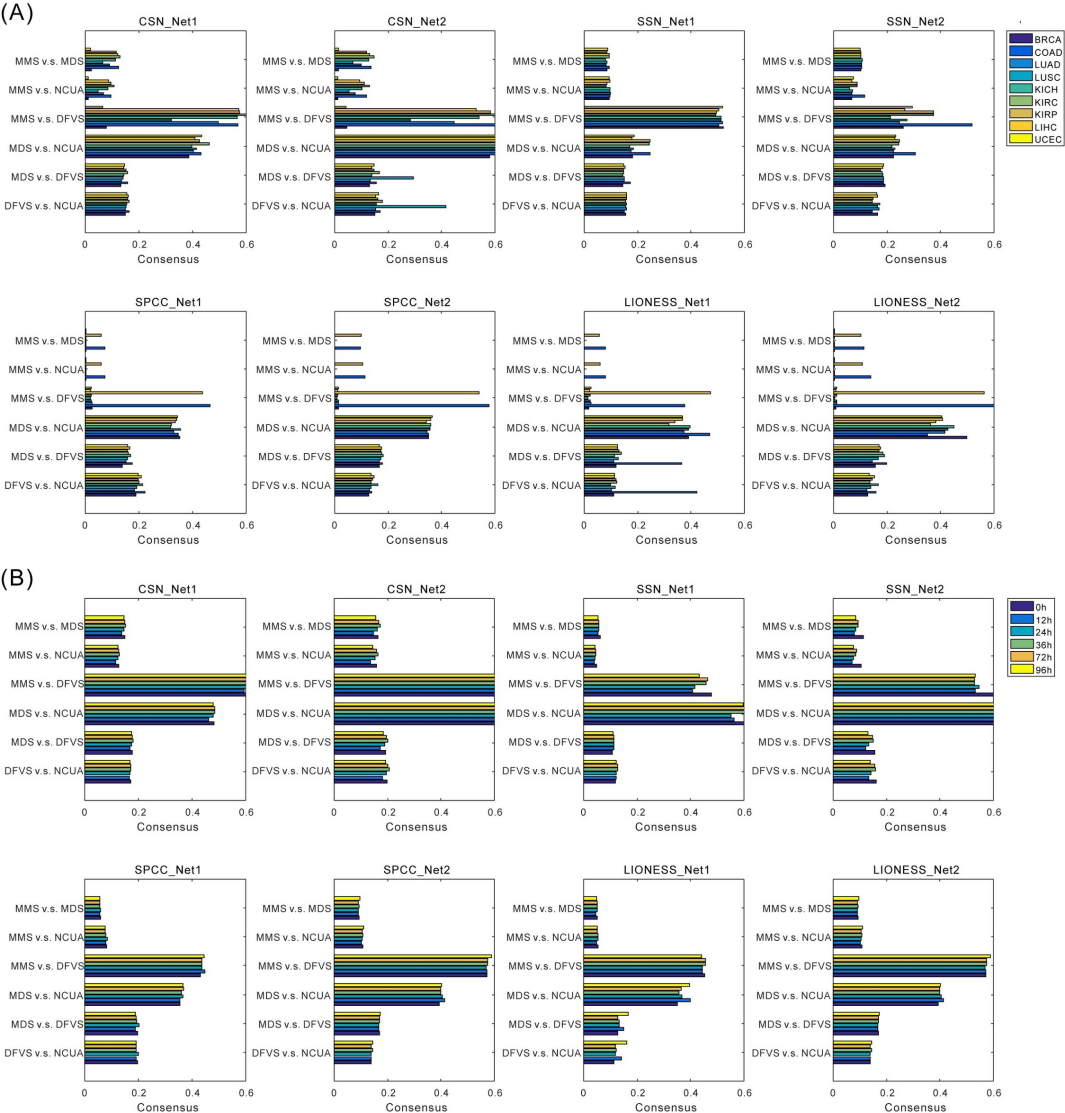
Evaluation of identification consensus among structural control methods. The comparisons are carried on any two control methods using (A) nine TCGA cancer datasets, and (B) temporal single cell datasets.

To evaluate whether different network structural control methods (down-stream of SSC analysis) can detect the same driver genes, the findings between any two control methods (e.g., MMS vs. MDS, MMS vs. DFVS, MMS vs. NCUA, MDS vs. DFVS, MDS vs. NCUA, and DFVS vs. NCUA) were compared by Jaccard scores (see **Materials and Methods**) between any two control methods as the method consensus. According to such consensus measurements of any two methods on nine cancer datasets (**Fig 9A**) and Chu-time dataset (**Fig 9B**), we found that the output similarity of two directed-network-based control methods (MMS_DFVS) and that of two undirected-network-based control methods (MDS_NCUA) indeed has a higher Jaccard score than other method pairs. These results suggest the type of network direction is an important factor to determine the method consensus. Besides, on the cancer datasets shown in **Fig 9A**, some paired structural methods (e.g., MMS vs. MDS, MMS vs. DFVS, and MMS vs. NCUA) actually showed the lowest consensus when SPCC and LIONESS were adopted, suggesting MMS performance might have more variations than other methods when the upstream sample-specific network construction methods approaches changed.

## Note 3: Robustness of network structural control methods for different reference networks

**Fig 10:**
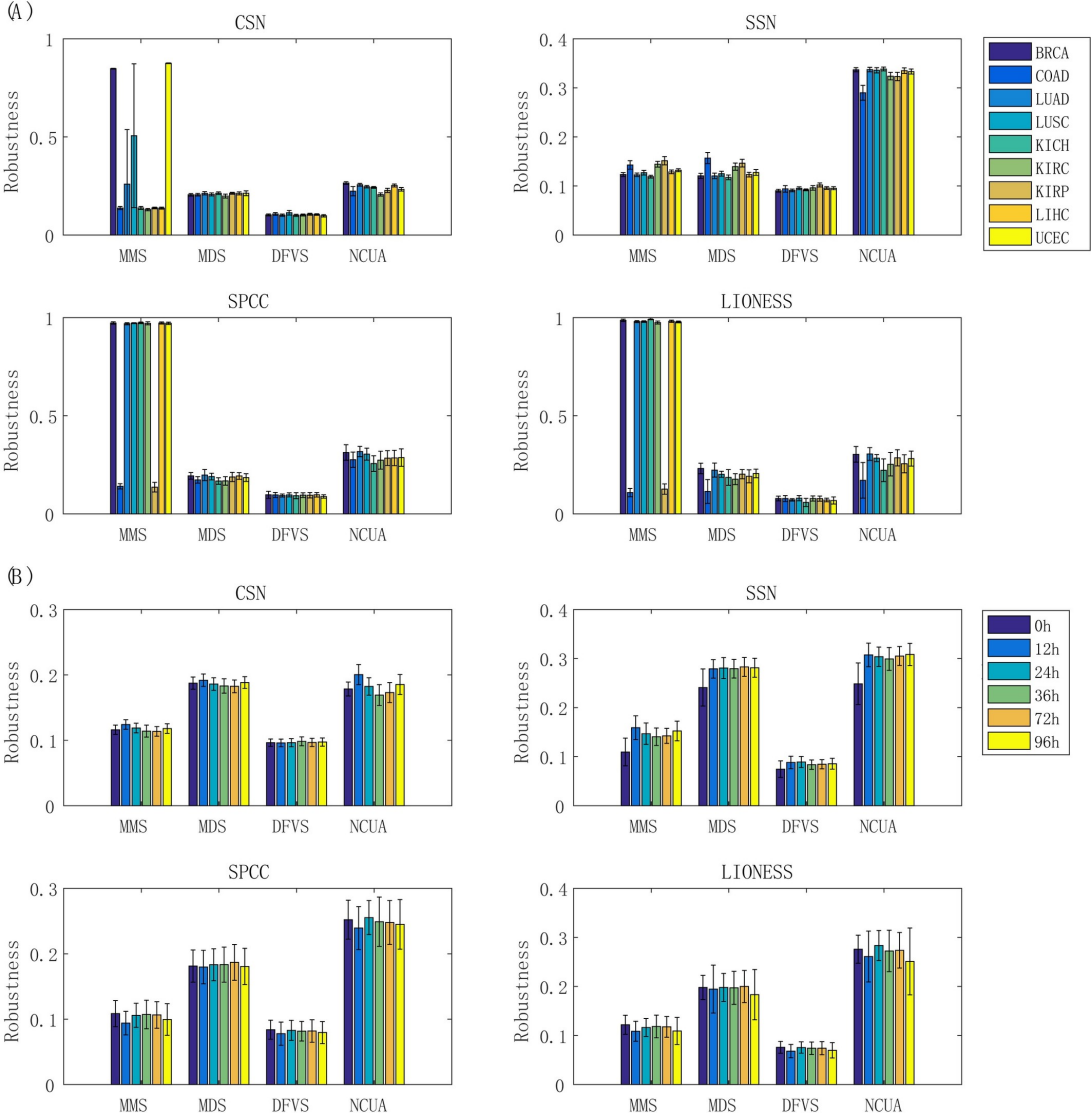
Evaluation of robustness of network structural control methods. The MMS, MDS, DFVS, and NCUA were compared, when two reference networks on CSN and SSN and SPCC and LIONESS used. The robustness of network structural control methods is shown in (A) for nine TCGA cancer datasets and in (B) for temporal single cell datasets.

Besides, to comprehensively evaluate the robustness of structural control methods when different reference networks were used, the results of a given control method were compared again by using two reference networks (e.g., MMS_Net1 vs. MMS_Net2, MDS_Net1 vs. MDS_Net2, DFVS_Net1 vs. DFVS_Net2, and NCUA_Net1 vs. NCUA _Net2), where the Jaccard score was applied as a measure of *robustness*. On the basis of the *robustness* scores of four network structural control methods on nine cancer datasets (**Fig 10A**) and Chu-time dataset (**Fig 10B),** all these methods would have relatively weak robustness when different reference networks were applied, independent of the exact dataset, which are also consistent to synthetic pinning control evaluations. In particular, on the cancer datasets, MMS and NCUA tended to have higher robustness than other methods (**Fig 10A**); meanwhile, on the Chu-time dataset, MDS and NCUA tended to outperform other methods (**Fig 10B**). Indeed, current network structural control methods are strongly dependent on the sample-specific network construction methods and are indeed sensitive to the underlying reference network structure. Therefore, the choice of a proper prior-given interaction network is a key for applying SSC analysis in research and applications.

**Fig 11:**
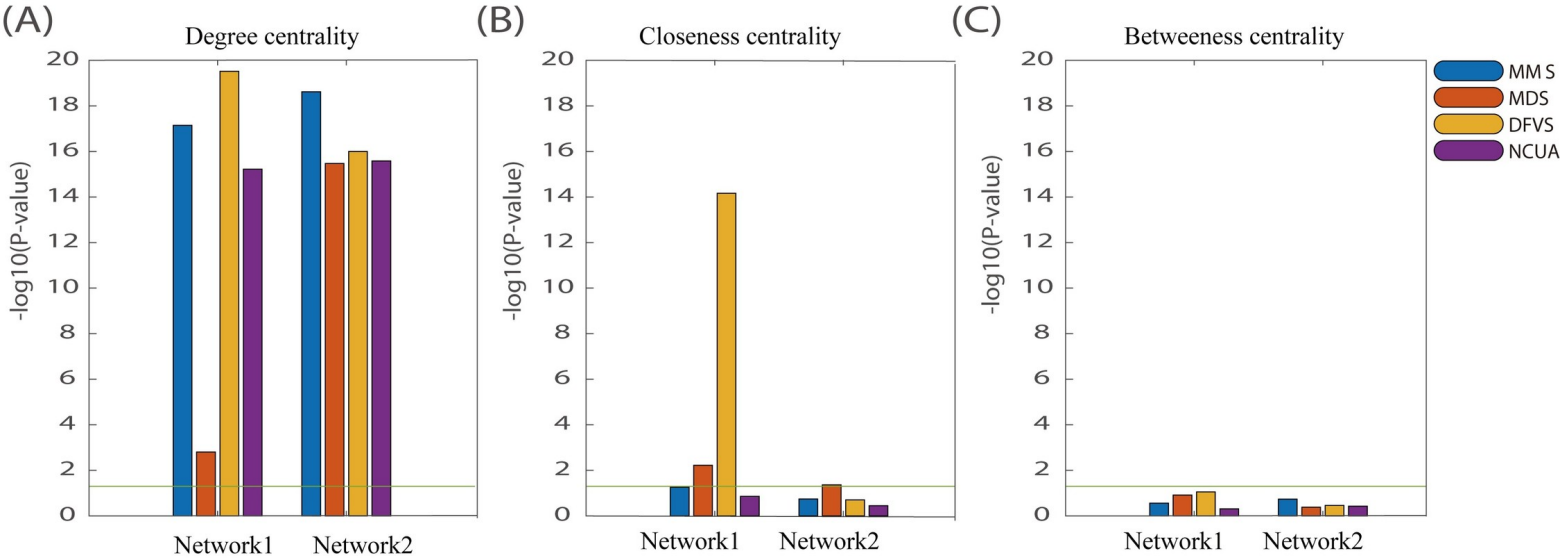
The *P*-value results of these network structure factors compared with random selection method. (A) Degree centrality; (B) Closeness centrality; (C) Betweeness centrality.

In addition, to investigate what network structure factors of reference network determine such performance differences, we evaluated the degree centrality, closeness centrality and betweeness centrality of network nodes. Similar to above strategy, we randomly selected the genes with the same number of sample-specific network control methods and we repeated this procedure 100 times; then we used a statistic test (3) to evaluate whether these network structure factors determine performance difference between SSC analysis and random selection. If *P*-value of such test is less than 0.05, we can regard that the network structure factors involved in network control methods is significantly different from those involved in the random selection method or others. As shown in **Fig 11**, the degree centrality and closeness centrality can significantly affect the network controllability which might serve as network structure factors to influence the performances of different methods, e.g. SSC workflows.

## Note 4: Driver configuration and network direction for different network structural control methods

**Fig 12:**
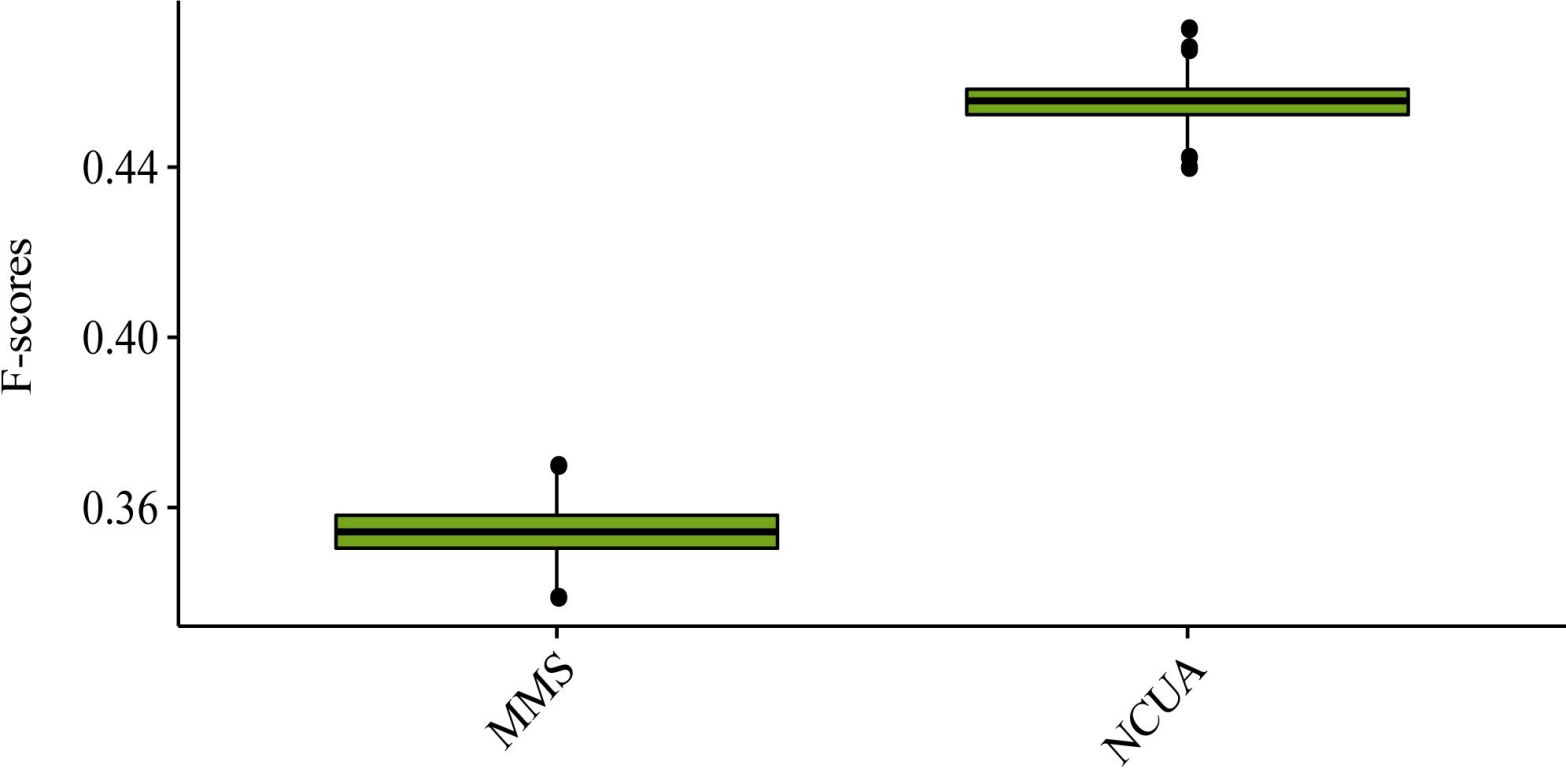
The F-score of the driver nodes’ enrichments in the list of cancer-associated genes in the human signaling network.

As above introduced, the network structural control methods are import down-stream work of SSC analysis workflow, whose driver configurations (i.e. identified driver node sets) are usually not unique especially for MMS, and remarkably related to network type (i.e. directed v.s. undirected). Finally, on a newly collected directed cell-signaling network [38,39], the performance of network control methods were compared again by considering the multiple driver nodes configurations. Considering that driver node sets are usually not unique in the directed cell-signaling network, a random markov sampling was applied to search the multiple sets of minimum driver nodes set for MMS [40] and NCUA (Material and Methods, and a diagram of the random markov sampling process for NCUA was illustrated in **Fig N in S1 File**). According to the F-score of the driver nodes’ enrichments in the list of cancer-associated genes[38,39]. the performance of NCUA was better than that of MMS (**Fig 12**). We should not that currently there are no theory model to find multiple driver node configurations for MDS and DFVS methods. It is worth studying how to find multiple driver node configurations for MDS and DFVS methods in the future, which will further benefit the applications of network control methods in biology.

## Discussion

In the last few years, many new algorithms for network structural control methods and sample-specific network construction methods have appeared to support a wide range of sample-specific biological data analysis using control principles. However, few of them had been compared directly, and guidelines and instructions are required to help develop and apply these cutting-edge approaches.

Single-sample network construction methods have been used to construct sample-specific interaction network for supporting identification of driver genes or drug targets or marker genes in biological networks by combining with network control methods, which can deepen our understanding on tumor or cell heterogeneity. To quantify the connections between network drivers identified by network theory, and driver genes or drug targets or marker genes in biology, we provided a performance evaluation of SSC analysis workflows based on the combination of four sample-specific network reconstruction methods and four representative network structural control methods on many bulk and single-cell expression dataset. By this study, we have shed light on the relative behavior and performance of different SSC implementations in particular application scenarios.

On one hand, for sample-specific network construction methods (i.e., the first step of SSC analysis), CSN or SSN are suggested as the preferred methods to try at first due to their competitive results compared to other methods; SPCC and LIONESS should be improved by considering more dynamic information from nonlinear association and matched controls in practice. On the other hand, for the network structural control methods (i.e., the second step of SSC analysis), MDS and NCUA could achieve higher efficiency, F-measure, and AUC scores than other methods on many datasets, although they do not currently use edge direction information for the identification of network driver nodes; MMS and DFVS would be secondary recommendations due to their potential to obtain meaningful results on particular datasets with high-quality edge direction information. Of note, when adopting a reference network as the prior-known part of CSN, SSN, SPCC, or LIONESS for obtaining high-confidence sample-specific state transition networks, it is suggested a few better-performing network structural control algorithms should be evaluated for analysis sensitivity.

Throughout this study, we have observed a number of issues that deserve to be points of focus in future investigations.

- The important network characteristics should be considered for SSC analysis in the future, including the network construction, prior-known network integration, and network direction determination and usage. For example, recently a partial correlation-based single-sample network (P-SSN) [41] retains the direct interactions by excluding indirect interactions and can provide new clues for constructing sample-specific networks. However, the P-SSN may generate more computational cost which should be considered for SSC analysis in the future.
- SSC mainly exploited dynamic analysis of node-networks from sample-specific network controllability perspective. However, recently edge-network dynamic analysis has become a hot topic in both sample-specific edge-network construction [42] and edge network controllability [43–47]. Therefore, how to extend SSC to the edge dynamic of sample-specific edge-network in biological application scenarios is an important direction in the future.
- Current SSC analysis ignored the temporal network dynamic information and would produce unnecessary false positives, thus SSC analysis should be shifted to temporal controllability so as to obtain more efficient and optimal controllers in the context of biological function and systems biology [48–50].
- Driver node sets are usually not unique and multiple driver node configurations are usually ignored for current SSC analysis. It is worth studying how to find multiple driver node configurations for SSC analysis, which will benefit the application of network control methods in diverse biology and biomedicine.

## Materials and methods

### The first step of SSC analysis: Sample-specific network construction methods

The CSN method is derived from a new theoretical model based on statistical dependency [5], which can be viewed as data transformation from the “unstable” gene expression data to the “stable” gene association data. CSN designs a statistic for genes *x*, *y* of sample *k* as:

$$\rho_{xy}^{k}=\frac{n_{xy}^{k}}{n}-\frac{n_{x}^{k}}{n}\frac{n_{y}^{k}}{n},$$ (4)

where $n_{x}^{k}=n_{y}^{k}=0.1n$ are predetermined integers (< *n*). In other words, CSN first draws the two boxes in data space near *x*_*k*_ and *y*_*k*_ based on the predetermined $n_{x}^{k}$ and $n_{y}^{k}$; then it can straightforwardly produce the third box, which is simply the intersection of the previous two boxes. Thus, CSN can obtain the value of $n_{xy}^{k}$ by counting the data sample plots in the third box, thereby testing the criterion of Eq (4). The original work has shown that if *x* and *y* are independent of each other, the statistic $\rho_{xy}^{k}$ approximately follows a normal distribution, and the mean value and standard deviation are:

$$\mu_{xy}^{k}=0,$$ (5)

$$\sigma_{xy}^{k}=\sqrt{\frac{n_{x}^{k}n_{y}^{k}(n-n_{x}^{k})(n-n_{x}^{k})}{n^{4}(n-1)}},$$ (6)

Therefore, for CSN, the edge between genes *x* and *y* is determined by the statistic (4) and the *P*-value corresponding to this statistic can be obtained from Eqs (5) and (6).

SSN is a statistical method to construct an individual-specific network solely based on expression data of a single sample [4], rather than the aggregated network for a group of samples, based on statistical perturbation analysis of a single sample against a group of given control samples. For the SSN method, the co-expression network of the tumor sample network or normal sample network for each patient is first constructed based on statistical perturbation analysis of one sample against a group of given reference samples (e.g., choosing the normal sample data of all of the patients as the reference data). If the differential Pearson Correlation Coefficient (Δ*PCC*) of an edge is significantly large based on the evaluation of the SSN method, this edge would be kept for the single sample (e.g. normal sample or tumor sample) from each patient. In detail, the *P*-value for an edge can be obtained from the statistical Z-value measuring Δ*PCC*. All the edges with significantly differential correlations (e.g., *P*-value < 0.05) are used to constitute the SSN for one normal sample or tumor sample. Finally, the personalized gene state transition network can be composed of selected edges existing in both the collected reference network and the referred differential co-expression network for each patient. In addition, the edge weight can be quantified by the differential fold change of gene pairs between the normal sample and the tumor sample for a given patient. Of note, the Δ*PCC* of an edge between genes *i* and *j* and its significant Z-score are calculated using the following formulas:

$$\begin{array}{l} \Delta PCC_{ij}=\left|PCC_{ij}^{n+1}-PCC_{ij}^{n}\right|\\ Z=\frac{\Delta PCC_{ij}}{\left(1-{\left(PCC_{ij}^{n}\right)}^{2})/(n-1\right)} \end{array},$$ (7)

where $PCC_{ij}^{n}$ is the *PCC* of an edge between genes *i* and *j* in the reference network with *n* samples; and $PCC_{ij}^{n+1}$ is the *PCC* of the edge between genes *i* and *j* in the perturbed network with one additional sample, given that this new sample (e.g. normal sample or tumor sample) for each patient is added to the reference sample group.

For the paired-SSN method [22], the co-expression network of the tumor sample network and normal sample network for each patient is constructed in the same way as for the SSN method. Then, the personalized differential co-expression network between the normal sample network and tumor sample network can be constructed in which the edge will exist if the *P*-value of the gene pair is less than (greater than) 0.05 in the tumor network but greater than (less than) 0.05 in the normal network for their corresponding patient. Note that by using SSN or paired SSN, the reference network can generally also be used to filter the noise in estimated co-expression networks.

To overcome the difficulty in obtaining correlations or edges from one sample, the SPCC approach [1,2] was first developed by decomposing each PCC measurement into multiple additive elements that form a new vector embedding correlation-like information of two variables for one sample. Then the transformation of gene correlation between gene *x* and gene *y* into edge space is carried out as follows:

$$SPCC_{ij}^{s}=\frac{x_{i}^{s}-\bar{x_{i}}}{\delta_{i}}\frac{x_{j}^{s}-\bar{x_{j}}}{\delta_{j}},$$ (8)

where $\bar{x_{i}}$ and *δ*_*i*_ are the mean and standard variance of the expression value of gene *i* in the group of *n* samples; and $x_{i}^{s}$ is the expression value of gene *i* in the sample *s*. To construct the sample-specific network, after we obtain the SPCC distribution *S* of all of the gene pairs we choose a threshold to determine the differential expression edges in the sample-specific network as follows:

w=μ(S)+2δ(S), (9)

where *μ*(*S*) and *δ*(*S*) are the mean and standard variance of the SPCC distribution *S* of all gene pairs.

LIONESS does not rely upon differential analysis between the tumor sample and a group of normal samples, and it reconstructs the individual specific network in a population of tumor samples as the personalized gene state transition network for each tumor sample [3]. LIONESS constructs the state transition network by calculating the edge statistical significance between all the tumor samples and the tumor samples without a given single sample. As LIONESS can be applied to multiple aggregate network reconstruction approaches, we used LIONESS applied to PCC to model the sample-specific state transition network in order to guarantee a fair comparison here. The network specific to one sample *s* in terms of the aggregate networks is then:

$$e_{ij}^{s}=N(e_{ij}^{N}-e_{ij}^{N-s})+e_{ij}^{N-s},$$ (10)

where $e_{ij}^{N}$ and $e_{ij}^{N-s}$ are the PCC values of gene pairs *i* and *j* in sample *n* and all but sample *s*, respectively; *N* is the number of the samples. Similar to the SPCC method, we use Eq (9) to determine the final differential expression edges in the sample-specific network.

### The second step of SSC analysis: Structure-based network control methods

For SSC problems, a sample-specific system is generally considered as the following broader model class:

dx/dt=F(x,A)+Bu, (11)

where *x*∈*R*^*N*×1^ denotes the state at time *t*, respectively, in the sample system, ***A***∈*R*^*N*×*N*^ represents the state transition matrix, and $B\in R^{N\times N_{C}}$ characterizes the driving by the ***N***_***C***_ controllers with the genes. The “controllers” in network control can produce input signals to realize the state transition of the whole network. The element to ***B***_***ij***_ is nonzero if the *j*-th input signal directly acts on node *v*_*i*_. The input matrices are set as $B^{T}=[I(b_{k_{1}}),Ι(b_{k_{2}}),\ldots ,I(b_{k_{T}})]$; $\left\{b_{1},b_{2},\ldots ,b_{N_{C}}\right\}$ are the indices of the set of constrained control genes ***U***, and *I*(*i*) denotes the *i*-_th_ column of the *N*×*N* identity matrix ***I***.

So far, the studies exploiting the structure-based control of complex networks can be mainly divided into two categories according to the styles of the network dynamics [51]. One is for linear dynamical networks; many researchers have developed structural control workflows, including the Maximum Matching Set (MMS)-based control methods [7] and the Minimum Dominating Set (MDS)-based control methods [9], the purpose of which is to identify the minimum number of input nodes that need to be controlled by external signals for the system to achieve the desired control objectives. The other is for nonlinear dynamical networks. Feedback Control workflows, such as the Directed FVS-based control method (DFVS) and the Nonlinear Control of Undirected networks Algorithm (NCUA), investigate the control of large networks in a reliable and nonlinear manner, where the network structure is prior known and the functional form of the governing equations is not specified, but must satisfy some continuous, dissipative, and decaying properties [10,11].

In the MMS-based and MDS-based control methods, a sample-specific network can be considered a system with the canonical linear time-invariant dynamics as follows:

dx/dt=Ax+Bu, (12)

For the MMS-based and MDS-based control methods, the minimum subset

*K* = {*K*_1_,*K*_2_,…,*K*_*T*_} of $\left\{b_{1},b_{2},\ldots ,b_{N_{C}}\right\}$ needs to be found to satisfy the following criterion:

$$max\left\{rank\left(\left[CB,CAB,CA^{2}B,\ldots ,CA^{N‐1}B\right]\right)\right\}=N_{O}$$ (13)

when Eq (13) is satisfied, the system in Eq (12) is structurally controllable. Note that the maximum in Eq (13) implies that given the input matrices ***B***, one needs to choose the proper nonzero weights in ***A*** to satisfy Eq (13). For MMS-based control methods, the driver nodes can be identified by using the maximum matching algorithm to ensure that there are no inaccessible nodes and no dilation that can guarantee the system is completely controllable [7]. Note that the MDS method focuses on the controllability study for undirected networks by assuming that each edge in a network is bidirectional [9]. That is, MDS assumes that the state transition matrix ***A*** in (11) is symmetric. The identification of MDS can be solved by using Integer Linear Programming (ILP) formalization as follows:

$$\begin{array}{l} \min\sum_{i\in V}x_{i}\\ s.t.x_{i}+\sum_{j\in \partial (i)}x_{j}\geq 1,x_{i},x_{j}\in \{0,1\} \end{array},$$ (14)

where ∂(*i*) denotes the neighborhood nodes of node *i*.

For this Feedback Control, F(*x*,*A*), the enhancement of the activity of the nodes in a sample-specific system, must satisfy: (i) the continuous differentiability of F(*x*,*A*), that is, F(*x*,*A*)∈*C*^1^, (ii) the dissipativity, that is, for any initial condition *x*(0) and for a finite time *t*≥0, the dynamic state *x*(*t*) is bounded by a positive constant *C*: ‖*x*_*n*_(*t*)‖≤*C*, and (iii) the decay condition ∂_1_F(*x*,*A*)<0, that is, the nonlinear dynamic function is not any nonlinear function but requires only a few conditions (e.g., continuous, dissipative, and decaying) on the nonlinear functions [10,11]. To drive the state of a network to any one of its naturally occurring end states (i.e., dynamical attractors), one needs to manipulate a set of nodes called Feedback Vertex Set (FVS) that intersects every feedback loop in the network, uniquely determining the long-term dynamics of the entire network. The definition of FVS is a subset of nodes in the graph, such that the removal of the set leaves the graph without feedback loops. Given a directed graph *G* = (*V*‚ *E*), the FVS can be calculated using ILP formalization [52]. This algorithm utilizes a scheme that enables weights to be assigned to vertices to capture an ordering relationship among the vertices. The ILP is formalized as follows:

$$\begin{array}{l} \min\sum_{i\in V}x_{i}\\ s.t.w_{i}-w_{j}+nx_{i}\geq 1(every\;v_{i}\to v_{j})\\ 0\leq w_{i}\leq 1,x_{i},x_{j}\in \{0,1\} \end{array},$$ (15)

Then the noted ILP can be used to perform the FVS calculation in the directed network.

Recently, DFVS was proposed under the framework of Feedback Control to study dynamic models of direct networks[21]. DFVS illustrates that controllability can be determined by the cycle structure and the source nodes of a directed network with FVS. DFVS, however, mainly focuses on the structural control of direct networks with nonlinear dynamics. Therefore, to solve the control problem of nonlinear undirected networks, Guo et al. developed the novel NCUA, under the framework of Feedback Control, which is based on the assumption that the edges of these undirected networks are modeled as bidirected edges [22]. For a given undirected network *G* (*V*, *E*), Guo et al. assumed that each edge was bidirectional, and *G* (*V*, *E*) was converted into a bipartite graph *G* (*V*_T_,*V*_⊥_,*E*_1_), where *V*_T_≡*V* and *V*_⊥_≡*E*. If *v*_*i*_*∈V*_T_ is one of the nodes for *v*_*j*_*∈V*_⊥_,they add an edge connecting *v*_*i*_ and *v*_*j*_ into set *E*_1_. After the bipartite graph is obtained, they adopt a modified version of the dominating set, in which the dominating set must be selected from *V*_T_ and is also sufficient to dominate all of the nodes in *V*_⊥_. The minimum dominating set cover problem can be solved by the following ILP model:

$$\begin{array}{l} \min\sum_{v\in V_{⊥}}x_{v}\\ s.t.,\sum_{\{v,u\}\in E_{1}}x_{v}\geq 1(every\;u\in V_{T}),x_{v}\in \{0,1\} \end{array},$$ (16)

where the variable *x*_*i*_ will take the value 1 when node *i* belongs to the cover set; the objective is to obtain the minimum number of nodes to cover set *V*_⊥_.

As noted, MDS, DFVS, and NCUA can be solved by ILP formulation. It is well known that the ILP problem is an NP-hard problem. However, the optimal solution can still be efficiently obtained for moderate sizes of graphs with up to a few tens of thousands of variables by utilizing an LP-based classic branch and bound method.

### Identification of multiple sets of minimum nodes set by using random markov sampling

To search the multiple sets of minimum node set, we designed a random Markov chain sampling strategy [40] to obtain different driver node sets. To use such strategy for NCUA, we defined a set of minimum dominating nodes in the “bipartite graph” as a Markov chain. The state space ***G*** of the Markov Chain (MC) is the set of all the possible minimum dominating nodes of the “bipartite graph”. The different MCs need to be sampled from the state space G so that a random MC method is used. The MC approach samples different sets of minimum dominating nodes randomly. Thus, the MC method can give different sets of driver nodes. The basic idea of the MC method for NCUA is to build a Markov Chain whose states are collections of the minimum dominating nodes in the top nodes covering the bottom nodes in the bipartite graph *G*(*V*_*T*_, *V*_⊥_, *E*_1_). As shown in **Fig N in S1 File**, we gave a diagram to illustrate the process of random markov sampling strategy for NCUA. The details were introduced below:

**Initialization:** By using ILP, obtain the initial Markov Chain *M*_*0*._

**Iteration:** For *t* = 1, 2,…, obtain *M*_t+1_ from *M*_*t*_ as follows:

- Choose a node *w* uniformly at random in *M*_t_. Then, delete node *w* and add a new node *vϵV*_*T*_−*M*_*t*_ which can cover the edges connected by node *w* in the bipartite graph *G*(*V*_*T*_, *V*_⊥_, *E*_1_). A new Markov Chain *M*_*t*+1_ = *M*_*t*_−{*w*}+{*v*} has been obtained.
- Accept the new Markov Chain *M*_t+1_ randomly.

### Assessment indices of SSC analysis

We used the following metrics of F-measure, AUC, controllability of sample, and Enrichment significance to assess the performance of SSC analysis workflows.

**F-measure:** To verify the effectiveness of the SSC analysis, the F-measure is considered to assess the enrichment ability of predicted control/driver nodes in a given gold standard list from biological or biomedical fields, which considers both the precision and the recall) using the formula:

$$F_{i}=2P_{i}R_{i}/(P_{i}+R_{i}),$$ (17)

where *P*_*i*_ denotes the fraction of correctly predicted genes among all of the predicted genes (precision) and *R*_*i*_ denotes the fraction) of correctly predicted personalized driver genes among the given dataset (recall).

**AUC:** To evaluate the usage efficiency of sample-specific network driver nodes for personalized drug discovery, we calculated the number of targeted sample-specific network driver nodes matching with drug combinations and rank anti-cancer drug combinations for each patient. The drug combinations annotated in the CAC drugs were applied to assess the AUC [53] of the top-ranked/predicted anti-cancer drug combinations from different SSC analysis workflows. We assigned a probability to each personalized gene according to its rank by using the formula:

$$p(CD_{j}^{i})=1-\frac{rank(CD_{j}^{i})}{CD^{i}}$$ (18)

where $CD_{j}^{i}$ denotes the predicted score of combinational drug *j* for sample *i* and *CD*^*i*^ denotes score of all the combinational drugs for sample *i*. Based on the predicted probability and the true label in the CAC, we can obtain the AUC value of predicted anti-cancer drug combinations when the prediction threshold shifts.

**Jaccard score:** To measure the consensus of different methods on the same dataset and the robustness of one method when two reference networks are used, we adopt the Jaccard score as follows:

$$S_{i}(m_{1},m_{2})=\frac{\left\|D_{i}(m_{1})\cap D_{i}(m_{2})\right\|}{\left\|D_{i}(m_{1})\cup D_{i}(m_{2})\right\|},$$ (19)

where *D*_*i*_(*m*_1_) and *D*_*i*_(*m*_2_) denote the driver node sets of sample *i* by using methods *m*_1_ and *m*_2_, respectively.

**Controllability of sample:** For each sample, we evaluated the *controllability* of each sample by structural control methods on the sample-specific state transition network, which is assumed to measure the power of each sample for changing its current state. The controllability is defined as:

$$Controllability_{i}=1-\frac{\left\|D_{i}\right\|}{\left\|G\right\|}$$ (20)

where *D*_*i*_ denotes the driver node set of sample *i* and *G* denotes the set of all of the genes. The bigger the *controllability* is, the greater the probability the sample state is capable of change.

**Enrichment significance:** To estimate the significant overlap between the driver predictions *S* and a given list *D*, we computed the *P*-value by the hypergeometric test [36] as follows:

$$P‐value=P(X\geq k)=\sum_{k=1}^{\infty }\frac{\left(\begin{array}{l} K\\ k \end{array}\right)\left(\begin{array}{l} N-K\\ n-k \end{array}\right)}{\left(\begin{array}{l} K\\ n \end{array}\right)},$$ (21)

where *N* is the number of all genes in the network, *K* is the number of a given gold standard list *D*, *n* is the number of the driver predictions *S*, and *k* is the number of the driver predictions overlapping with the genes in *D*. If the enrichment *P*-value for one method’s output/prediction is less than 0.05, we regard this method to have satisfactory driver predictions when using the prior-known gold standard.

## Supporting information

S1 File Some supplementary tables and figures in the manuscript. **Table A in S1 File: Sample information in TCGA Cancer datasets. Each individual has paired samples (control sample and tumor sample) Fig A in S1 File. Precision of structural control methods for driver genes identification on 9 TCGA bulk cancer data sets.** By using (A-B) CSN_Net1 and CSN_Net2, (C-D) SSN_Net1 and SSN_Net2, (E-F) SPCC_Net1 and SPCC_Net2 and (G-H) LIONESS_Net1 and LIONESS_Net2, we can obtain different state transition network. Therefore we respectively evaluate the performance (Precision) of 4 structure control methods on these different state transition networks. **Fig B in S1 File. Recall of structural control methods for driver genes identification on 9 TCGA bulk cancer data sets.** By using (A-B) CSN_Net1 and CSN_Net2, (C-D) SSN_Net1 and SSN_Net2, (E-F) SPCC_Net1 and SPCC_Net2 and (G-H) LIONESS_Net1 and LIONESS_Net2, we can obtain different state transition network. Therefore we respectively evaluate the performance (Recall) of 4 structure control methods on these different state transition networks. **Fig C in S1 File. The heatmap in terms of the average rate of F-scores in the new SSC corresponding to the old SSC from all reference samples on Network-1. Fig D in S1 File. The heatmap in terms of the average rate of F-scores in the new SSC corresponding to the old SSC from all reference samples on Network-2. Fig E in S1 File. The heatmap in terms of the average rate of F-scores of network control methods on BRCA cancer patient data with network deconvolution corresponding to those without network deconvolution method on (A) Network-1 and (B) Network-2. Fig F in S1 File. Evaluation of structural control methods using the efficiency number, i.e., the fraction of cells enriched in factor genes that are involved in human embryonic development at each time point.** Different methods were used for these different state transition networks, including (A) CSN_Net1, (B) CSN_Net2, (C) SSN_Net1, (D) SSN_Net2, (E) SPCC_Net1, (F) SPCC_Net2, (G) LIONESS_Net1, and (H) LIONESS_Net2. **Fig G in S1 File. For nine TCGA data set, identification of ‘Dark genes’ by using different methods on these different state transition networks including** (A-B) CSN_Net1 and CSN_Net2, (C-D) SSN_Net1 and SSN_Net2, (E-F) SPCC_Net1 and SPCC_Net2 and (G-H) LIONESS_Net1 and LIONESS_Net2. **Fig H in S1 File. For *Chu-time data set*, identification of “Dark-differential expression genes”by using different methods** on these different state transition networks including (A-B) CSN_Net1 and CSN_Net2, (C-D) SSN_Net1 and SSN_Net2, (E-F) SPCC_Net1 and SPCC_Net2 and (G-H) LIONESS_Net1 and LIONESS_Net2. **Fig I in S1 File. (A1-A4) The z-score results of SSC analysis control for enriching in CGC genes on BRCA cancer data compared with random selection method.**(A1) CSN, (A2) SSN, (A3) LIONESS, and (A4) SPCC. **(B1-B4) The z-score results of SSC analysis control for enriching in CGC genes on BRCA cancer data compared with degree-preserved random selection method.**(B1) CSN, (B2) SSN, (B3) LIONESS, and (B4) SPCC. **Fig J in S1 File. (A1-A4) The z-score results of SSC analysis control for enriching in clinical efficient combinational drugs on LUAD cancer data compared with random selection method.**(A1) CSN, (A2) SSN, (A3) LIONESS, and (A4) SPCC. **(B1-B4) The z-score results of SSC analysis control for enriching in clinical efficient combinational drugs on LUAD cancer data compared with degree-preserved random selection method.**(B1) CSN, (B2) SSN, (B3) LIONESS, and (B4) SPCC. **Fig K in S1 File. (A1-A4) The z-score results of SSC analysis control for enriching in dark genes on BRCA cancer data compared with random selection method.**(A1) CSN, (A2) SSN, (A3) LIONESS, and (A4) SPCC. **(B1-B4) The z-score results of SSC analysis control for enriching in dark genes on BRCA cancer data compared with degree-preserved random selection method.**(B1) CSN, (B2) SSN, (B3) LIONESS, and (B4) SPCC. **Fig L in S1 File. The *significance score* results of SSC analysis control for enriching in 20 human embryonic differentiation functional genes on Chu-time single cell data compared with random selection method.** (A) CSN_Net1, (B) CSN_Net2, (C) SSN_Net1, (D) SSN_Net2, (E) LIONESS_Net1, (F) LIONESS_Net2, (G) SPCC-Net1, and (H) SPCC_Net2. **Fig M in S1 File. The *significance score* results of SSC analysis control for enriching in 20 human embryonic differentiation functional genes on Chu-time single cell data compared with degree-preserved random selection method.** (A) CSN_Net1, (B) CSN_Net2, (C) SSN_Net1, (D) SSN_Net2, (E) LIONESS_Net1, (F) LIONESS_Net2, (G) SPCC-Net1, and (H) SPCC_Net2. **Fig N in S1 File. A schematic diagram illustrating the markov chaining for NCUA algorithm.** We assume that each edge in a network is bi-directional and construct a bipartite graph from the original undirected network, in which the nodes of top side are the nodes of original graph and the nodes of the bottom side are the edges of the original graph. Then, we adapt an equivalent optimization procedure for obtaining the initial input nodes *M*_1_ = {*v*_1_,*v*_4_,*v*_9_} as a initial Markov chain within the top side nodes to cover the bottom side nodes in the bipartite graph that are sufficient to control the whole network with nonlinear dynamics in mathematical term. Finally, we generate a new Markov chain *M*_2_ = {*v*_1_,*v*_8_,*v*_9_} by replacing node *v*_4_ with node *v*_8_ in the Markov chain *M*_1_ which can also cover edge and generated the new Markov chain *M*_2_ and repeat this process until the terminated condition is satisfied.
(DOCX)

S2 File The interactions between combinatorial drugs and targets from multiple datasets. (XLSX)

S3 File The drug combinations annotated in the Clinical Anti-cancer Combinational drugs. (XLSX)

S4 File The functional genes that are involved in human embryonic differentiation. (XLSX)

S5 File The list of dark genes within dark gene regions from a canonical dataset. (XLSX)
