# Supplementary material for: Performance assessment of sample-specific network control methods for bulk and single-cell biological data analysis
Source: PLoS Comput Biol. 2021 May 6;17(5):e1008962. doi: 10.1371/journal.pcbi.1008962 (PMC8130943; doi:10.1371/journal.pcbi.1008962)
Supplement: S1 File — Table A in S1 File: Sample information in TCGA Cancer datasets. Each individual has paired samples (control sample and tumor sample) Fig A in S1 File. Precision of structural control methods for driver genes identification on 9 TCGA bulk cancer data sets. By using (A-B) CSN_Net1 and CSN_Net2, (C-D) SSN_Net1 and SSN_Net2, (E-F) SPCC_Net1 and SPCC_Net2 and (G-H) LIONESS_Net1 and LIONESS_Net2, we can obtain different state transition network. Therefore we respectively evaluate the performance (Precision) of 4 structure control methods on these different state transition networks. Fig B in S1 File. Recall of structural control methods for driver genes identification on 9 TCGA bulk cancer data sets. By using (A-B) CSN_Net1 and CSN_Net2, (C-D) SSN_Net1 and SSN_Net2, (E-F) SPCC_Net1 and SPCC_Net2 and (G-H) LIONESS_Net1 and LIONESS_Net2, we can obtain different state transition network. Therefore we respectively evaluate the performance (Recall) of 4 structure control methods on these different state transition networks. Fig C in S1 File. The heatmap in terms of the average rate of F-scores in the new SSC corresponding to the old SSC from all reference samples on Network-1. Fig D in S1 File. The heatmap in terms of the average rate of F-scores in the new SSC corresponding to the old SSC from all reference samples on Network-2. Fig E in S1 File. The heatmap in terms of the average rate of F-scores of network control methods on BRCA cancer patient data with network deconvolution corresponding to those without network deconvolution method on (A) Network-1 and (B) Network-2. Fig F in S1 File. Evaluation of structural control methods using the efficiency number, i.e., the fraction of cells enriched in factor genes that are involved in human embryonic development at each time point. Different methods were used for these different state transition networks, including (A) CSN_Net1, (B) CSN_Net2, (C) SSN_Net1, (D) SSN_Net2, (E) SPCC_Net1, (F) SPCC_Net2, (G) LIONESS_Net1, and (H) LIO [file pcbi.1008962.s001.docx]

Supplementary manuscript of

**Performance assessment of sample-specific network control methods for bulk and single-cell biological data analysis**

Wei-Feng Guo^1,2^, Xiangtian Yu^3^, Qian-Qian Shi^4^, Jing Liang^1*^, Shao-Wu Zhang^2*^, Tao Zeng^5*^

^1^ School of Electrical Engineering, Zhengzhou University, Zhengzhou, China,

^2^ Key Laboratory of Information Fusion Technology of Ministry of Education, School of Automation, Northwestern Polytechnical University, Xian, China

^3^ Shanghai Jiao Tong University Affiliated Sixth People’s Hospital, Shanghai, China

^4^ Hubei Key Laboratory of Agricultural Bioinformatics, College of Informatics, Huazhong Agricultural University, Wuhan, China

^5^ CAS Key Laboratory of Computational Biology, Bio-Med Big Data Center, Shanghai Institute of Nutrition and Health, University of Chinese Academy of Sciences, Chinese Academy of Sciences, Shanghai, China.

* [liangjing@zzu.edu.cn (JL)](mailto:liangjing@zzu.edu.cn%20(JL));

* [zhangsw@nwpu.edu.cn](mailto:zhangsw@nwpu.edu.cn) (SWZ);

* [zengtao@sibs.ac.cn](mailto:zengtao@sibs.ac.cn) (TZ)

**Supplementary note 1: some other supplementary tables in the manuscript**

**Table A in S1 File: Sample information in TCGA Cancer datasets.** **Each individual has paired samples (control sample and tumor sample)**

| Abbreviation | Description | Number of paired samples |
| --- | --- | --- |
| BRCA | Breast invasive carcinoma | 112 |
| COAD | Colon adenocarcinoma | 50 |
| KICH | Kidney Chromophobe | 23 |
| KIRC | Kidney renal clear cell carcinoma | 72 |
| KIRP | Kidney renal papillary cell carcinoma | 31 |
| LIHC | Liver hepatocellular carcinoma | 50 |
| LUAD | Lung adenocarcinoma | 57 |
| LUSC | Lung squamous cell carcinoma | 49 |
| UCEC | Uterine Corpus Endometrial Carcinoma | 23 |

**Supplementary note 2: some other supplementary figures in the manuscript**


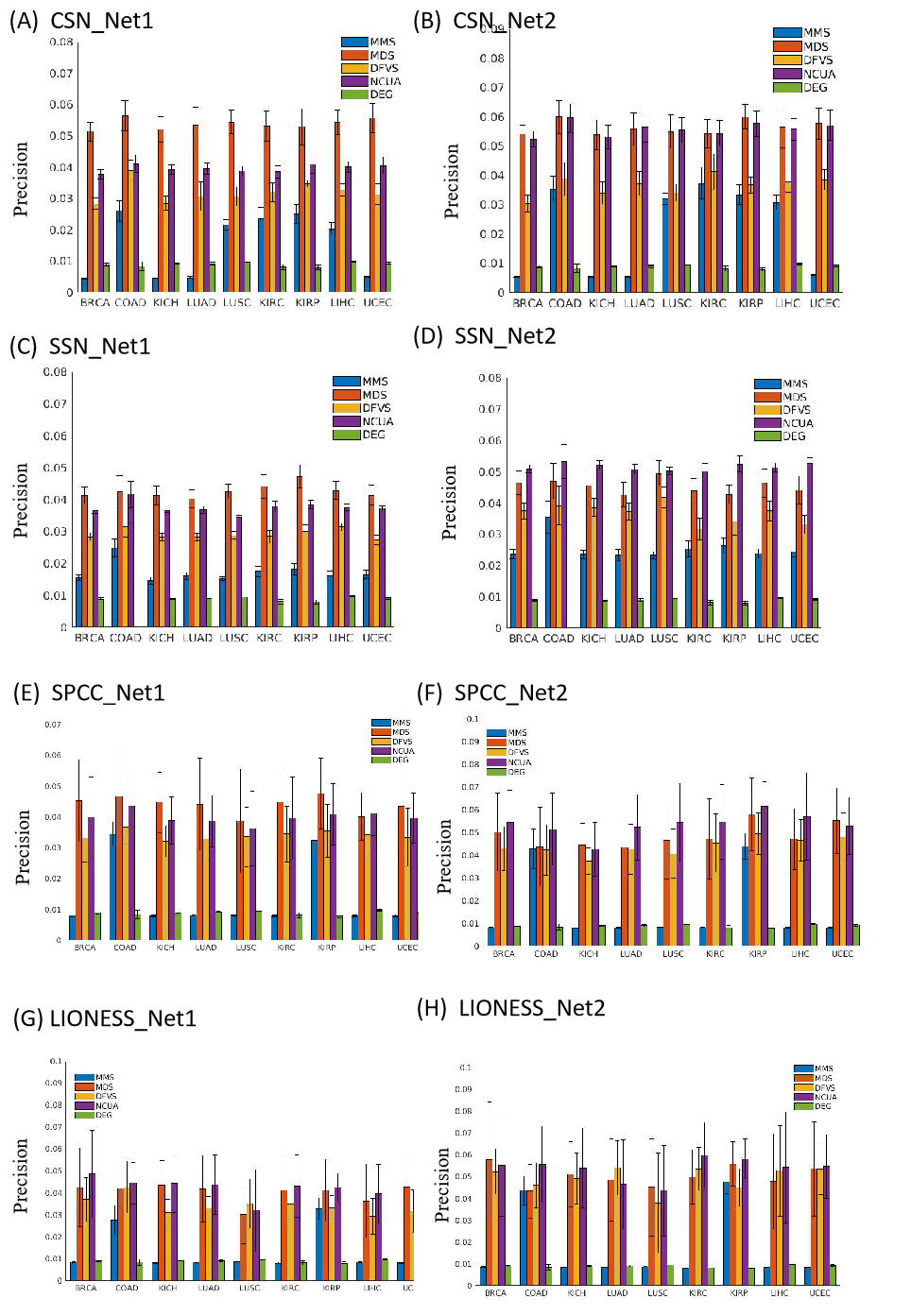


**Fig A in S1 File. Precision of structural control methods for driver genes identification on 9 TCGA bulk cancer data sets.** By using (A-B) CSN_Net1 and CSN_Net2, (C-D) SSN_Net1 and SSN_Net2, (E-F) SPCC_Net1 and SPCC_Net2 and (G-H) SPCC_Net1 and SPCC_Net2, we can obtain different state transition network. Therefore we respectively evaluate the performance (Precision) of 4 structure control methods on these different state transition networks.


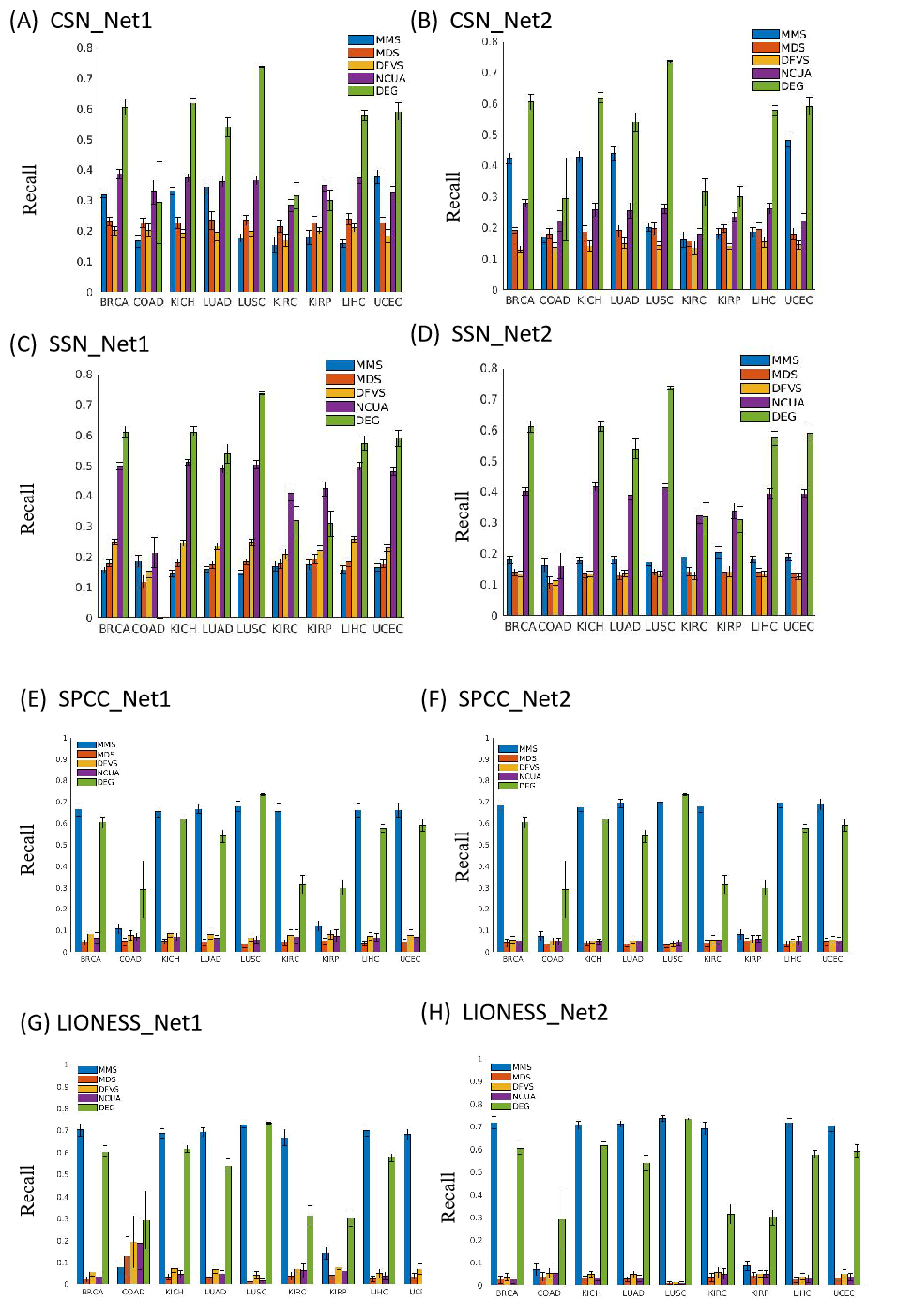


**Fig B in S1 File. Recall of structural control methods for driver genes identification on 9 TCGA bulk cancer data sets.** By using (A-B) CSN_Net1 and CSN_Net2, (C-D) SSN_Net1 and SSN_Net2, (E-F) SPCC_Net1 and SPCC_Net2 and (G-H) SPCC_Net1 and SPCC_Net2, we can obtain different state transition network. Therefore we respectively evaluate the performance (Recall) of 4 structure control methods on these different state transition networks.


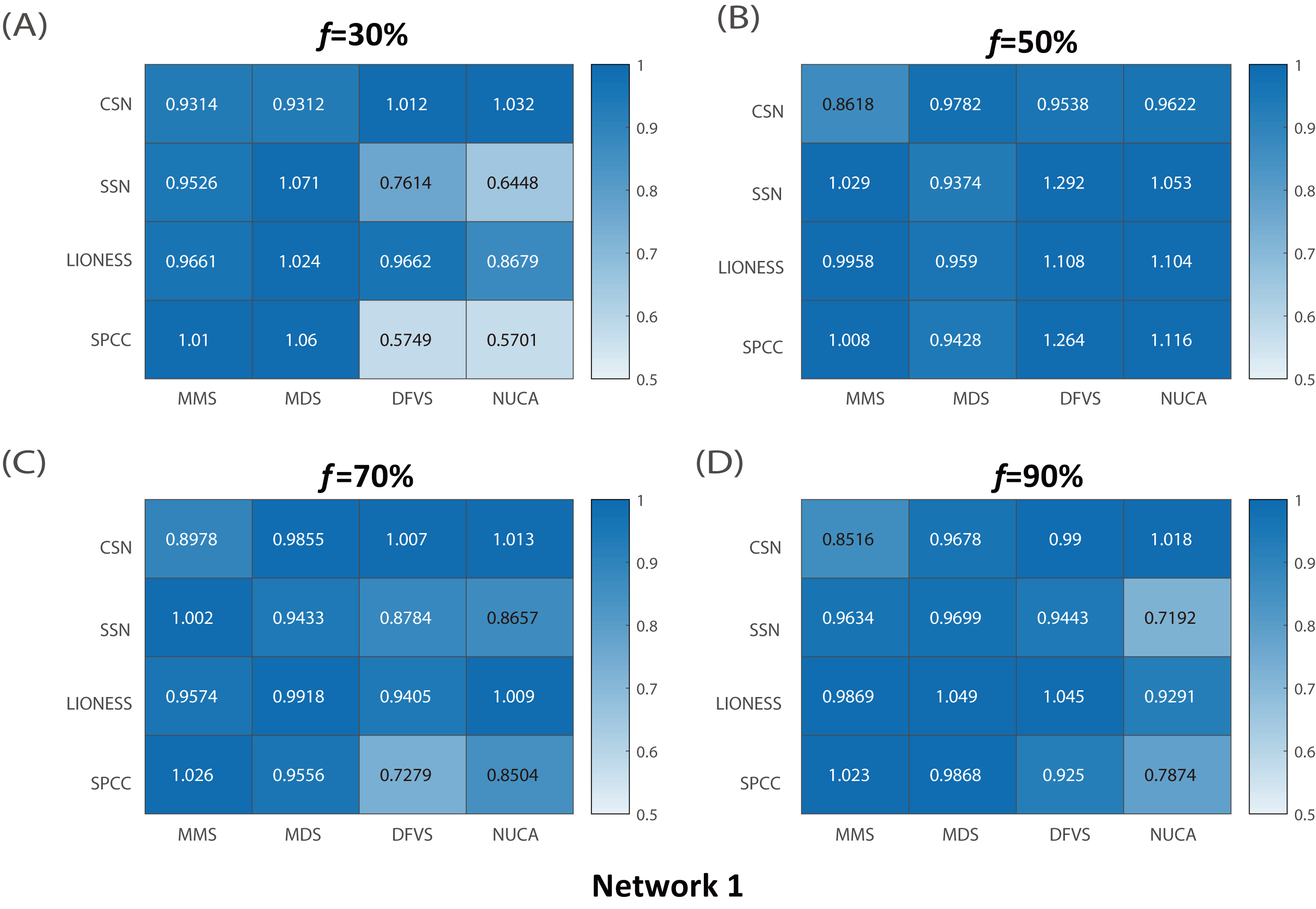


**Fig C in S1 File. The heatmap in terms of the average rate of F-scores in the new SSC corresponding to the old SSC from all reference samples on network 1.**


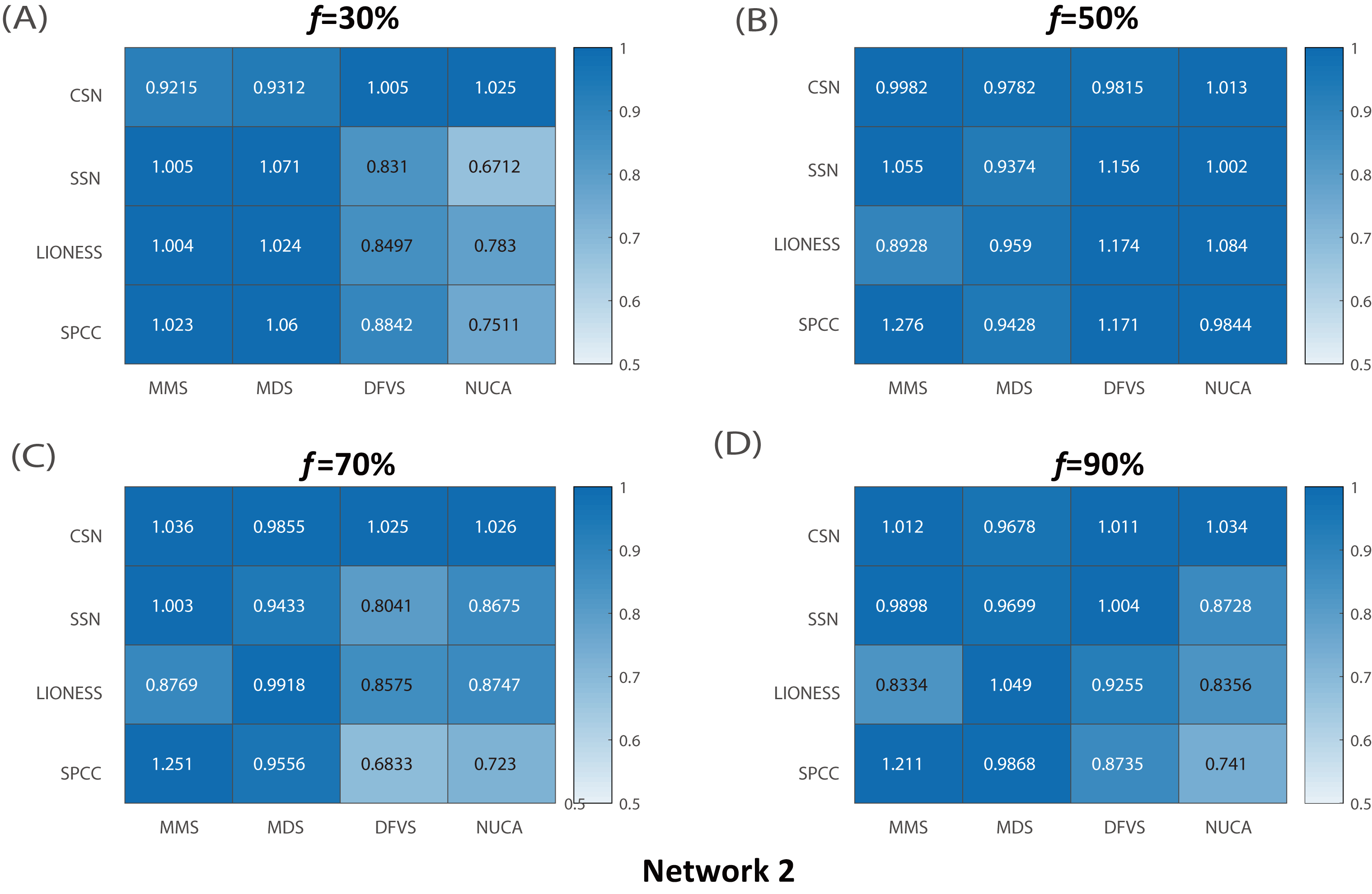


**Fig D in S1 File. The heatmap in terms of the average rate of F-scores in the new SSC corresponding to the old SSC from all reference samples on network 2.**


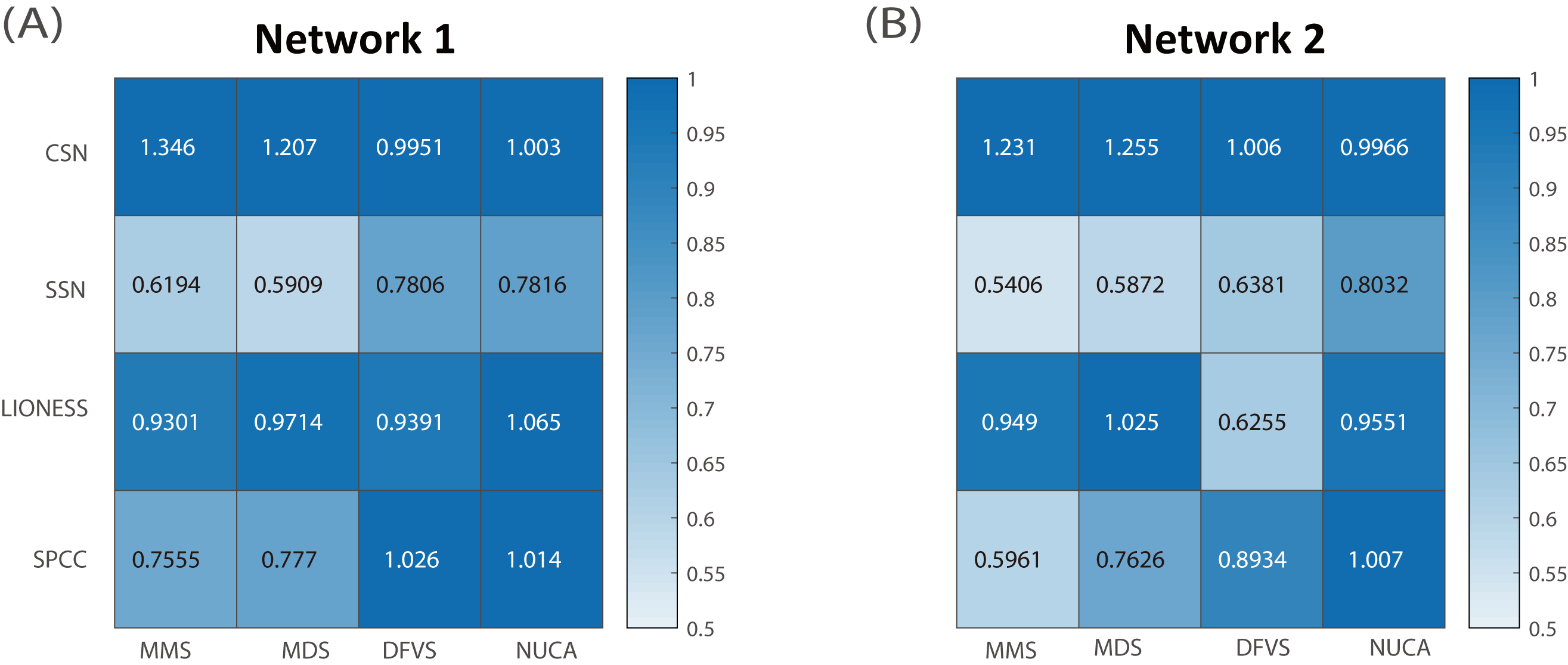


**Fig E in S1 File. The heatmap in terms of the average rate of F-scores of network control methods on BRCA cancer patient data with network deconvolution corresponding to those without network deconvolution method on (A) network 1 and (B) network 2.**


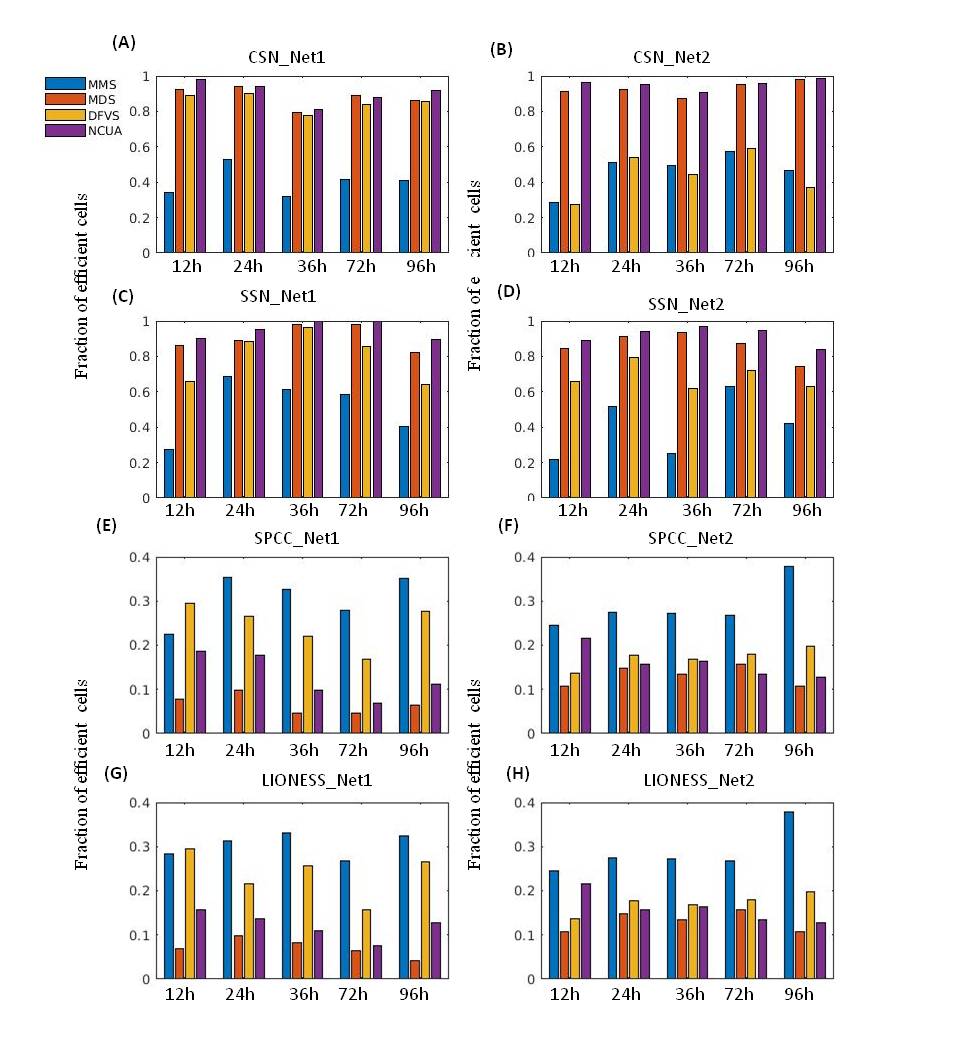


**Fig F in S1 File. Evaluation of structural control methods using the efficiency number, i.e., the fraction of cells enriched in factor genes that are involved in human embryonic development at each time point.** Different methods were used for these different state transition networks, including (A) CSN_Net1, (B) CSN_Net2, (C) SSN_Net1, (D) SSN_Net2, (E) SPCC_Net1, (F) SPCC_Net2, (G) LIONESS_Net1, and (H) LIONESS_Net2.

**
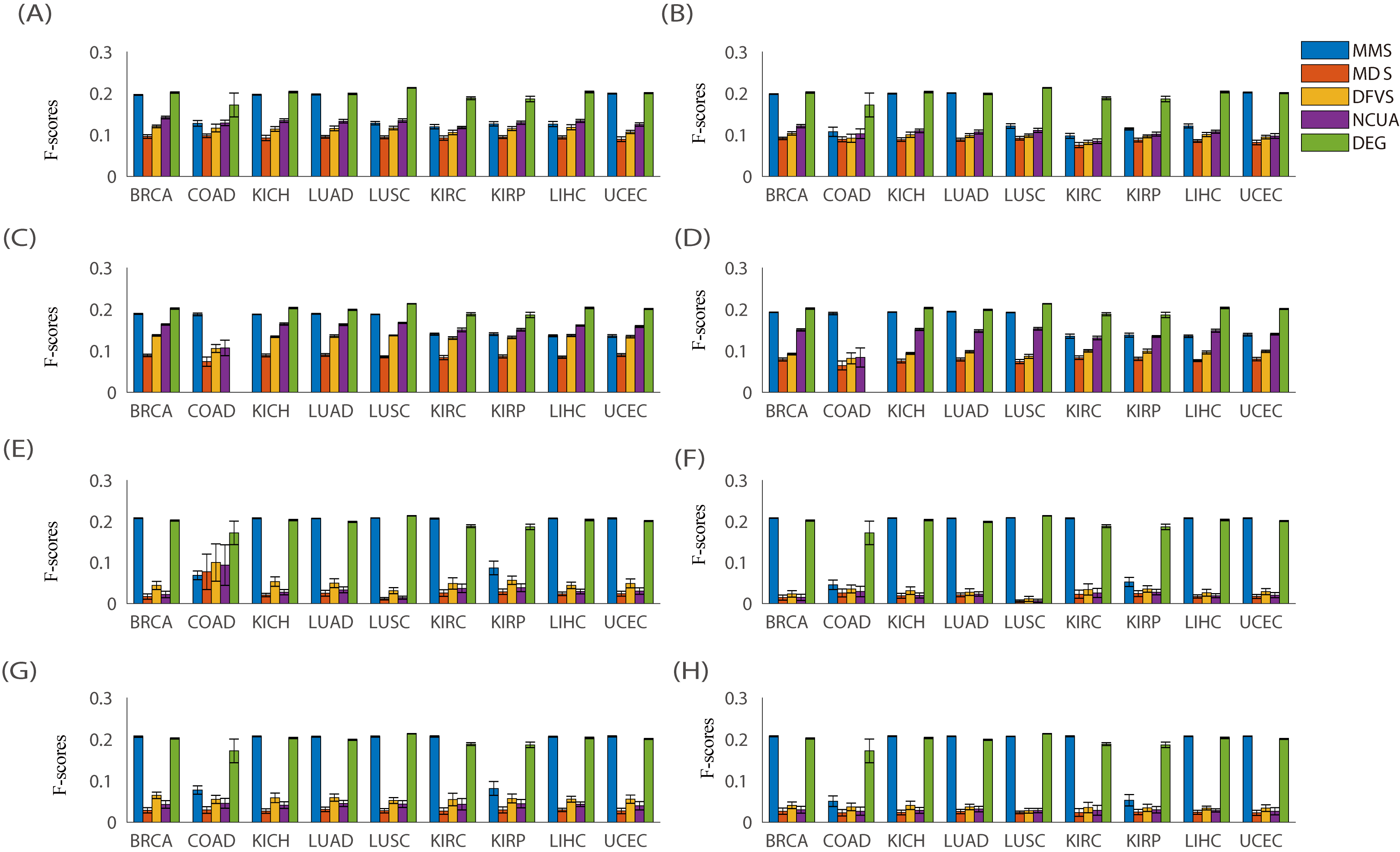
**

**Fig G in S1 File. For nine TCGA data set, identification of ‘Dark genes’ by using different methods on these different state transition networks including** (A-B) CSN_Net1 and CSN_Net2, (C-D) SSN_Net1 and SSN_Net2, (E-F) SPCC_Net1 and SPCC_Net2 and (G-H) LIONESS_Net1 and LIONESS_Net2.


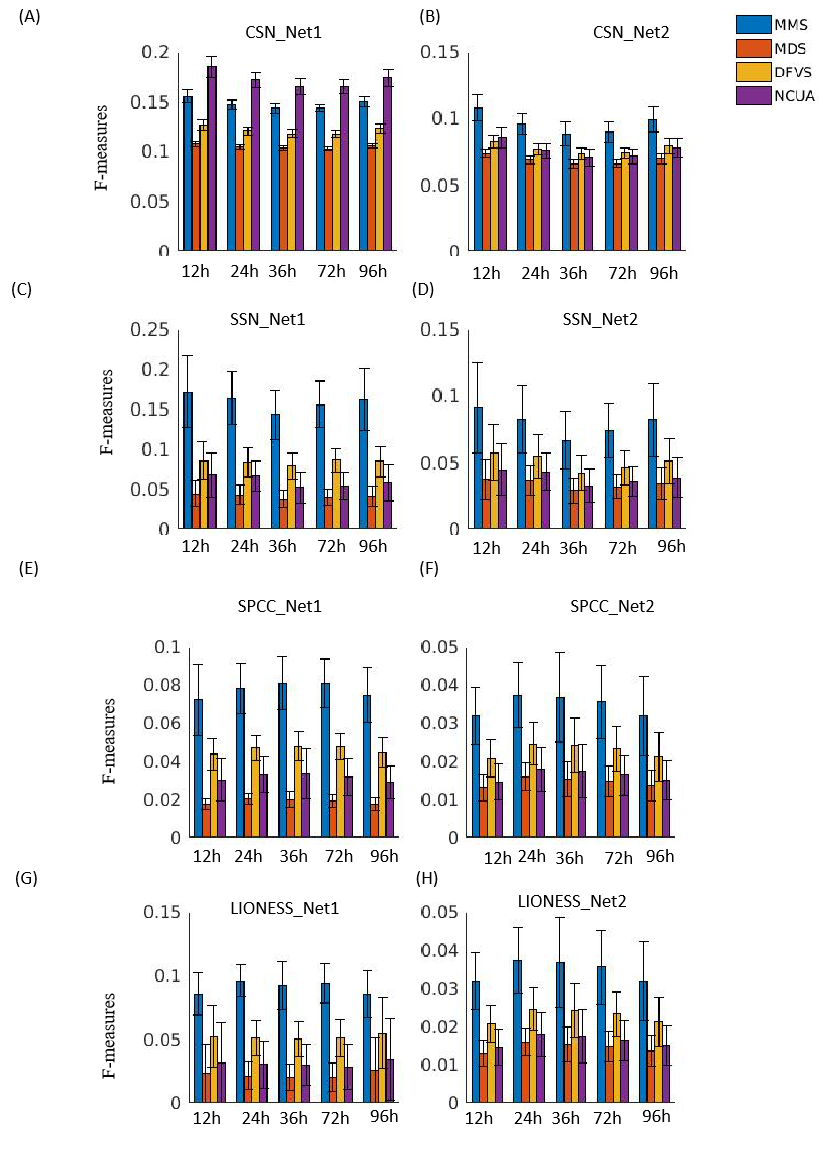


**Fig H in S1 File. For *Chu-time data set*, identification of “Dark-differential expression genes“ by using different methods** on these different state transition networks including (A-B) CSN_Net1 and CSN_Net2, (C-D) SSN_Net1 and SSN_Net2, (E-F) SPCC_Net1 and SPCC_Net2 and (G-H) LIONESS_Net1 and LIONESS_Net2.

*
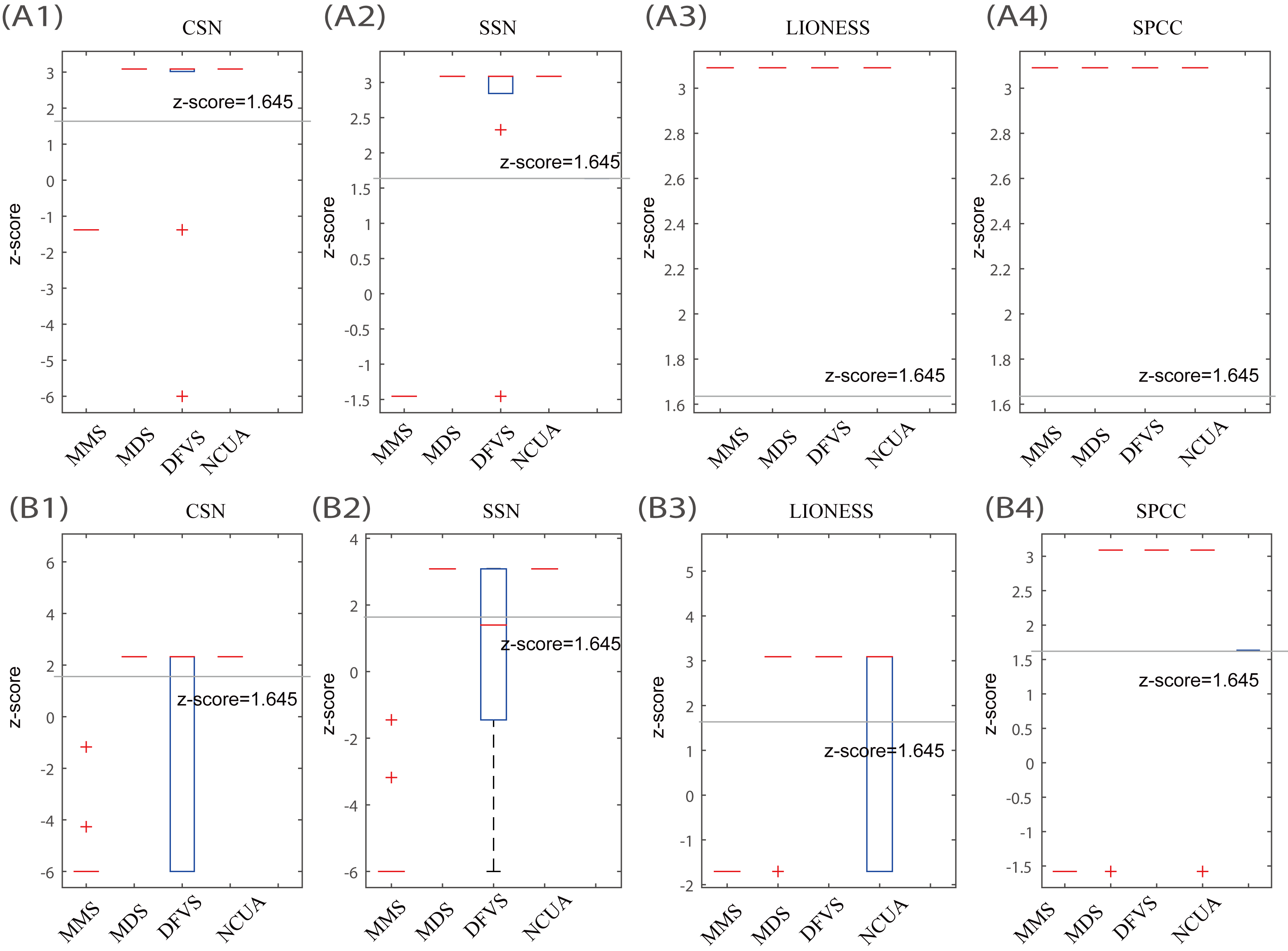
*

**Fig I in S1 File. (A1-A4) The z-score results of SSC analysis control for enriching in CGC genes on BRCA cancer data compared with random selection method.**(A1) CSN , (A2) SSN, (A3) LIONESS, and (A4) SPCC . **(B1-B4) The z-score results of SSC analysis control for enriching in CGC genes on BRCA cancer data compared with degree-preserved random selection method.**(B1) CSN , (B2) SSN, (B3) LIONESS, and (B4) SPCC .

*
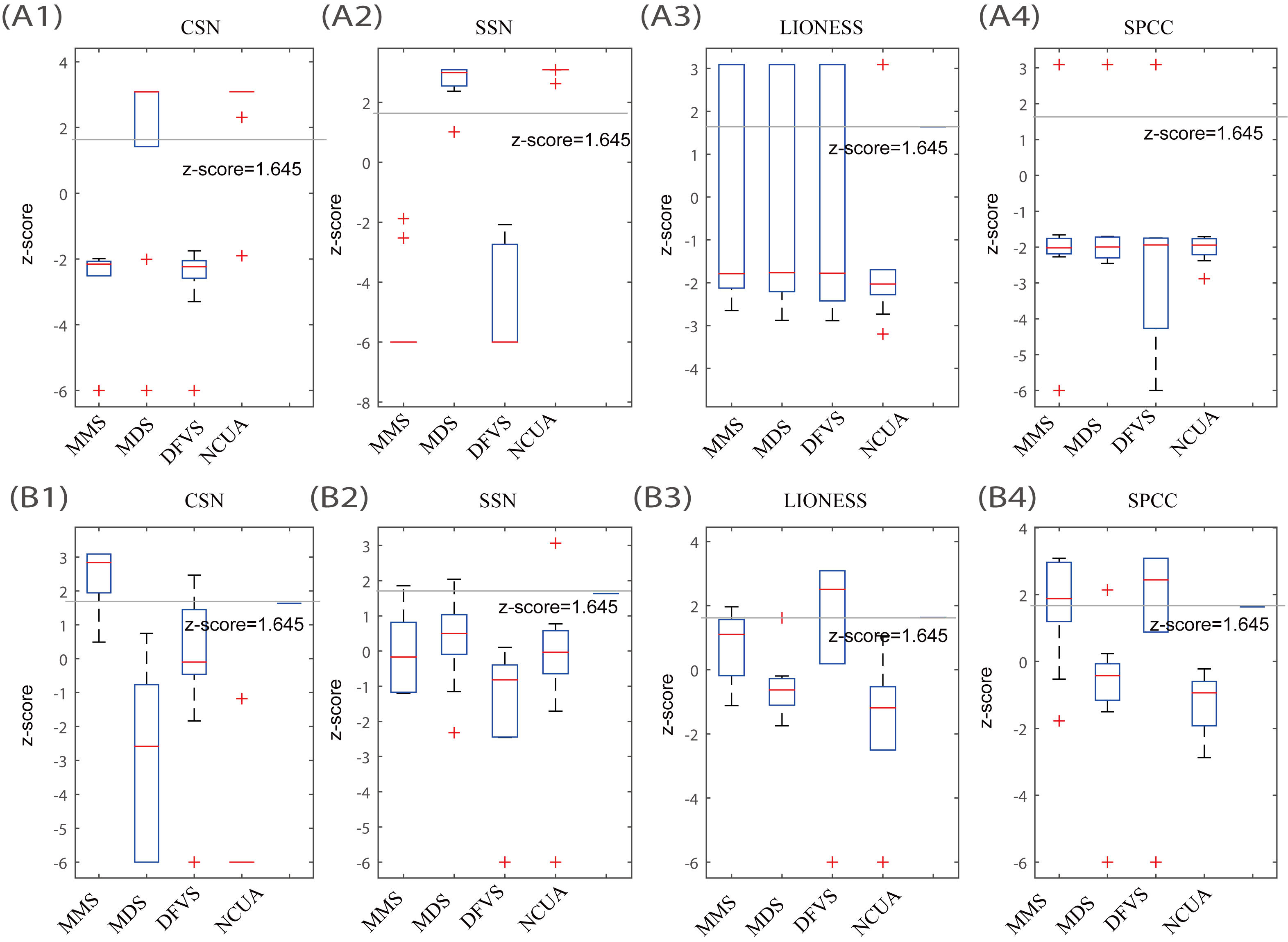
*

**Fig J in S1 File. (A1-A4) The z-score results of SSC analysis control for enriching in clinical efficient combinational drugs on LUAD cancer data compared with random selection method.**(A1) CSN , (A2) SSN, (A3) LIONESS, and (A4) SPCC . **(B1-B4) The z-score results of SSC analysis control for enriching in clinical efficient combinational drugs on LUAD cancer data compared with degree-preserved random selection method.**(B1) CSN , (B2) SSN, (B3) LIONESS, and (B4) SPCC .

*
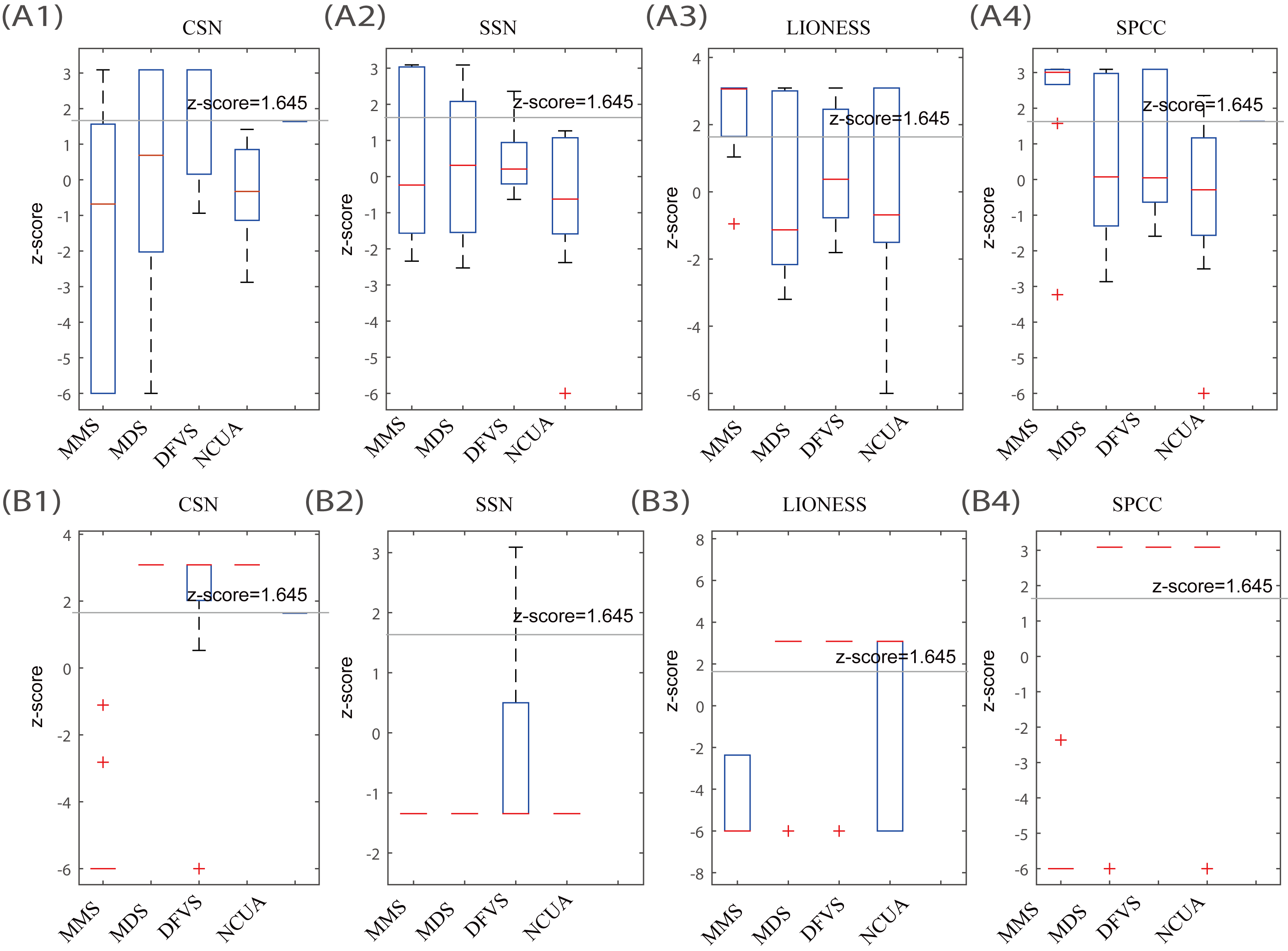
*

**Fig K in S1 File. (A1-A4) The z-score results of SSC analysis control for enriching in dark genes on BRCA cancer data compared with random selection method.**(A1) CSN , (A2) SSN, (A3) LIONESS, and (A4) SPCC . **(B1-B4) The z-score results of SSC analysis control for enriching in dark genes on BRCA cancer data compared with degree-preserved random selection method.**(B1) CSN , (B2) SSN, (B3) LIONESS, and (B4) SPCC .

*
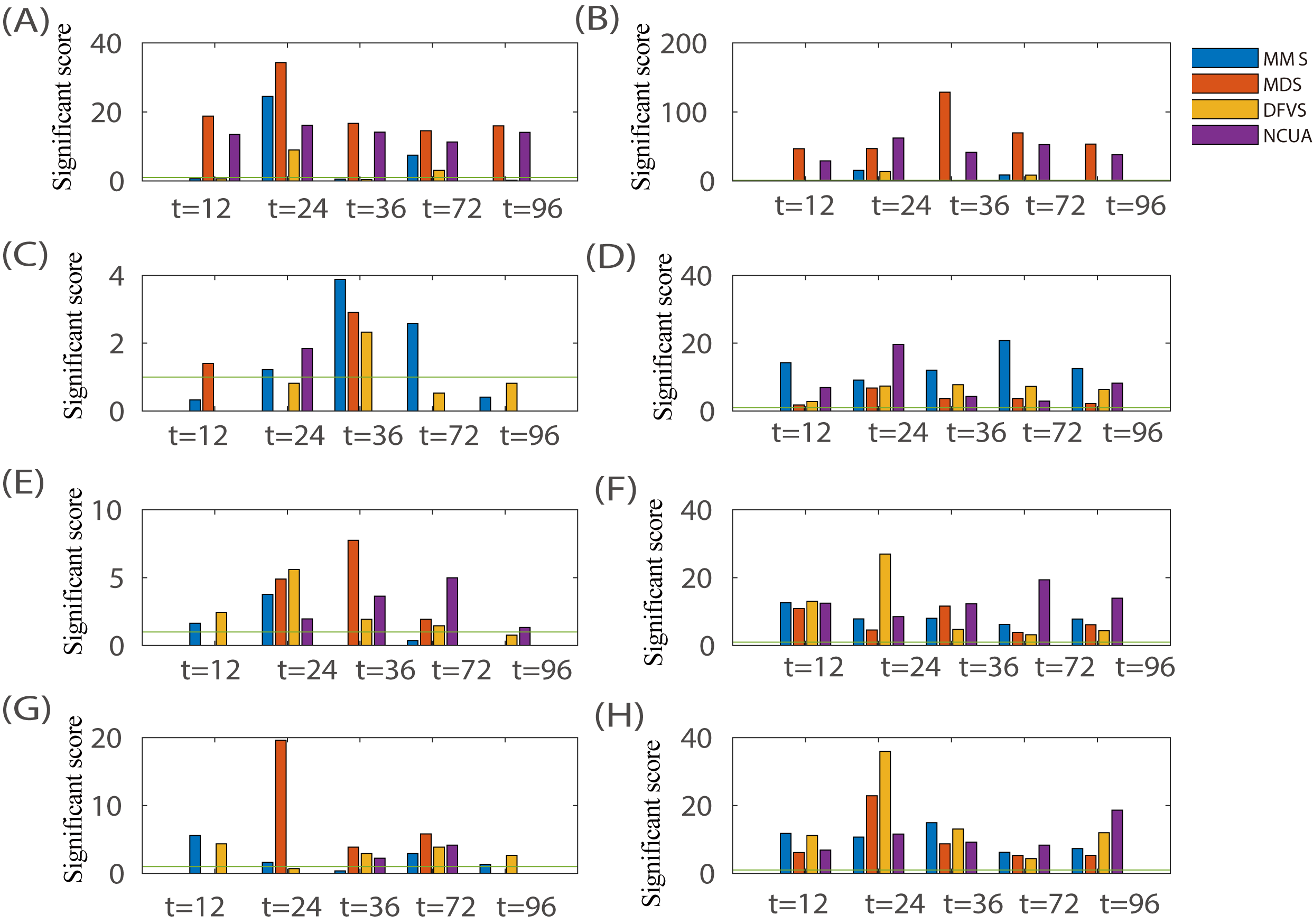
*

**Fig L in S1 File. The *significance score* results of SSC analysis control for enriching in 20 human embryonic differentiation functional genes on Chu-time single cell data compared with random selection method.** (A) CSN_Net1, (B) CSN_Net2, (C) SSN_Net1, (D) SSN_Net2, (E) LIONESS_Net1, (F) LIONESS_Net2, (G) SPCC-Net1, and (H) SPCC_Net2 .

*
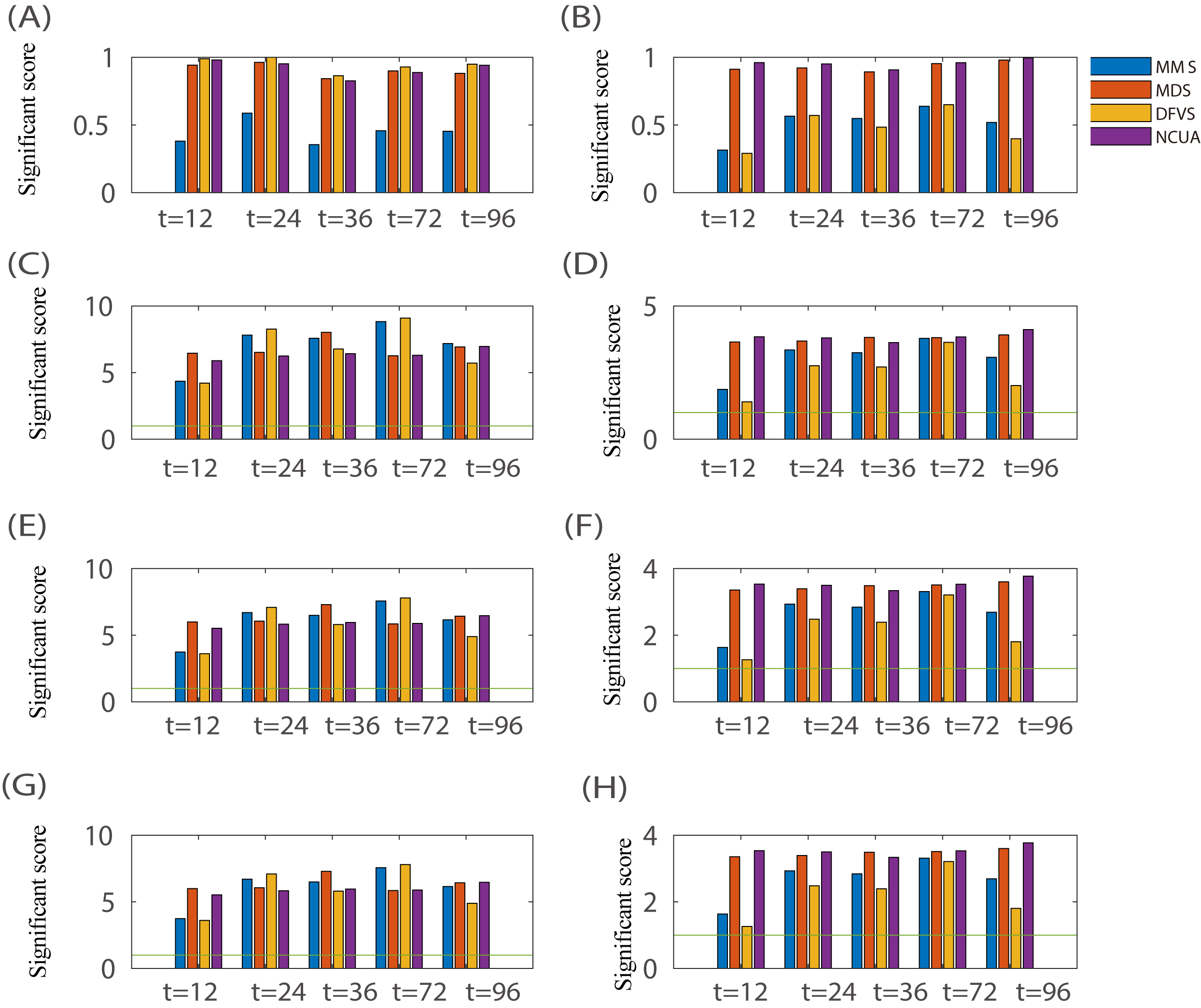
*

**Fig M in S1 File. The *significance score* results of SSC analysis control for enriching in 20 human embryonic differentiation functional genes on Chu-time single cell data compared with degree-preserved random selection method.** (A) CSN_Net1, (B) CSN_Net2, (C) SSN_Net1, (D) SSN_Net2, (E) LIONESS_Net1, (F) LIONESS_Net2, (G) SPCC-Net1, and (H) SPCC_Net2 .

**Fig N in S1 File. A schematic diagram illustrating the markov chaining for NCUA.** We assume that each edge in a network is bi-directional and construct a bipartite graph from the original undirected network, in which the nodes of top side are the nodes of original graph and the nodes of the bottom side are the edges of the original graph. Then, we adapt an equivalent optimization procedure for obtaining the initial input nodes *M*_1_ = {*v*_1_,*v*_4_,*v*_9_} as a initial Markov chain within the top side nodes to cover the bottom side nodes in the bipartite graph that are sufficient to control the whole network with nonlinear dynamics in mathematical term. Finally, we generate a new Markov chain *M*_2_ = {*v*_1_,*v*_8_,*v*_9_} by replacing node *v*_4_ with node *v*_8_ in the Markov chain *M*_1_ which can also cover edge and generated the new Markov chain *M*_2_ and repeat this process until the terminated condition is satisfied.
